# Plant-Climate Interaction Effects: Changes in the Relative Distribution and Concentration of the Volatile Tea Leaf Metabolome in 2014–2016

**DOI:** 10.3389/fpls.2019.01518

**Published:** 2019-11-22

**Authors:** Nicole Kfoury, Eric R. Scott, Colin M. Orians, Selena Ahmed, Sean B. Cash, Timothy Griffin, Corene Matyas, John Richard Stepp, Wenyan Han, Dayuan Xue, Chunlin Long, Albert Robbat

**Affiliations:** ^1^Department of Chemistry, Tufts University, Medford, MA, United States; ^2^Sensory and Science Center, Medford, MA, United States; ^3^Department of Biology, Tufts University, Medford, MA, United States; ^4^Department of Health and Human Development, Montana State University, Bozeman, MT, United States; ^5^Friedman School of Nutrition Science and Policy, Tufts University, Boston, MA, United States; ^6^Department of Geography, University of Florida, Gainesville, FL, United States; ^7^Department of Anthropology, University of Florida, Gainesville, FL, United States; ^8^Tea Research Institute, Chinese Academy of Agricultural Sciences, Hangzhou, China; ^9^College of Life and Environmental Sciences, Minzu University of China, Beijing, China

**Keywords:** climate change, plant-climate interactions, season, elevation, tea quality, metabolomics, gas chromatography/mass spectrometry

## Abstract

Climatic conditions affect the chemical composition of edible crops, which can impact flavor, nutrition and overall consumer preferences. To understand these effects, we sampled tea (*Camellia sinensis* (L.) Kuntze) grown in different environmental conditions. Using a target/nontarget data analysis approach, we detected 564 metabolites from tea grown at two elevations in spring and summer over 3 years in two major tea-producing areas of China. Principal component analysis and partial least squares-discriminant analysis show seasonal, elevational, and yearly differences in tea from Yunnan and Fujian provinces. Independent of location, higher concentrations of compounds with aromas characteristic of farmers’ perceptions of high-quality tea were found in spring and high elevation teas. Yunnan teas were distinct from Fujian teas, but the effects of elevation and season were different for the two locations. Elevation was the largest source of metabolite variation in Yunnan yet had no effect in Fujian. In contrast seasonal differences were strong in both locations. Importantly, the year-to-year variation in chemistry at both locations emphasizes the importance of doing multi-year studies, and further highlights the challenge farmers face when trying to produce teas with specific flavor/health (metabolite) profiles.

## Introduction

Greenhouse gasses accumulating in the atmosphere cause variability in local climates including prolonged heatwaves, droughts, heavy rains, flooding, cold, and frost, all of which damage crop production (IPCC, 2014; USGCRP, 2017; Fussel et al., 2018). The effect of extreme weather and climate variability on yield has been extensively studied for many crops (Kang et al., 2009; Lobell and Gourdji, 2012; Kurukulasuriya and Rosenthal, 2013). Most studies focus on one climate variable at a time; for example, temperature increases of up to 4°C are expected to decrease rice (Asia), wheat (India), and maize (US and Africa) yields by 20%–60% (NRC, 2011). Equally important to climate effects on yields is understanding how plant–climate interactions affect crop quality, including the secondary metabolites that contribute to the sensory and nutritional properties of plant materials. Changes in crop quality have been shown to influence consumer preference, acceptance, and, ultimately demand (Ahmed and Stepp, 2016). As attribution science (the science of attributing specific adverse weather conditions to climate change) advances (Otto, 2016; Imada et al., 2018; Knutson et al., 2018), understanding how plant–climate interactions influences human and natural systems is critical in developing long-term, sustainable agro-ecosystems.

Tea (*Camellia sinensis* (L.) Kuntze) is a long-lived crop grown in subtropical regions that are vulnerable to climate variability and is predicted to be severely impacted by climate change (IPCC, 2014; Han et al., 2018). The arrival of the East Asian Monsoon rains, an extreme weather event, is occurring earlier, lasting longer and shortening the spring harvest for high quality tea (Ahmed et al., 2014; Kowalsick et al., 2014; Boehm et al., 2016). Since tea plants are mostly grown in mountainous areas, elevational differences can also affect tea quality: higher quality generally occurs at higher elevation (Han et al., 2017; Kfoury et al., 2018b). Therefore, it is of great interest to understand the effect of season and elevation over multiple years in order to develop strategies for sustaining high quality tea in the era of climate change (FAO, 2015; Han et al., 2017; Han et al., 2018). Our previous work on tea in Yunnan Province, China reveals striking changes in the distribution and concentration of metabolites in response to differences in precipitation and temperature within a 1-year period (Ahmed et al., 2014; Kowalsick et al., 2014; Robbat Jr et al., 2017; Kfoury et al., 2018a; Kfoury et al., 2018b).

Tea has a complex secondary metabolite profile comprised of hundreds of volatile and non-volatile compounds that contribute to quality by impacting flavor, nutritional, and health attributes (Ahmed and Stepp, 2012). Of the ∼450 volatile metabolites detected in any given tea sample, our previous research found that two-thirds increased or decreased in concentration; most by more than 50%, more than 100 by 100%, and some by 1,000% due to differences in spring and summer rainfall (∼0 vs. 400 mm) and temperature (22°C vs. 28°C) as well as elevational differences in temperature (5°C) (Kowalsick et al., 2014; Kfoury et al., 2018b). We found a greater number of metabolites with higher concentrations in the spring (cooler temperature/no rain) and high elevation (cooler temperature) teas that exhibited sweet, floral, honey-like aromas compared to hay, grassy, earthy notes in the summer (warmer temperature/monsoon rain) and low elevation (warmer temperature) teas. These results are consistent with farmer sensory perceptions of high- and low-quality tea (Ahmed and Stepp, 2012; Ahmed et al., 2014; Han et al., 2017). High elevation teas also contained more and higher concentrations of volatile metabolites that possess analgesic, antianxiety, antibacterial, anticancer, antidepressant, antifungal, anti-inflammatory, antioxidant, antistress, and cardioprotective properties (Kfoury et al., 2018b). This finding is extremely important since time and location of harvest can affect the medicinal properties of tea extracts used in clinical trials (Tsao et al., 2009; Wang et al., 2014; Yan et al., 2017; Balsan et al., 2019).

Less dramatic, although equally important, are the differences in catechin (polyphenolic antioxidants) and methylxanthine (stimulants) concentrations. Although these metabolites were higher in spring tea, the monsoon rains induced higher total phenolic concentrations and antioxidant potential in summer tea (Ahmed et al., 2014). Also, the low elevation tea contained higher concentrations of caffeine, epicatechin gallate, gallocatechin, and catechin, while other catechins and methylxanthines were indifferent to elevation (Kfoury et al., 2018b). Importantly, many tea famers experience up to a 50% decrease in revenue for summer and low elevation tea most likely due to an increase in unpleasant aromatics and compounds associated with bitterness and astringency (Ahmed et al., 2014; Han et al., 2017).

Clearly, both elevation and season have important effects on the chemistry and quality of tea. Whether these differences hold true across years is understudied. Here, we performed a multi-year study to explore the effects of these drivers on tea quality. Specifically, we study the variation of tea quality based on the differences in metabolite distribution and concentration in tea due to seasonal and elevation differences over a 3-year period in Yunnan and Fujian Provinces, China. To understand how changes in climate will affect plant volatiles, it is necessary to obtain the total, detectable metabolome. Here, we employed a targeted/untargeted data analysis approach to analyze gas chromatography/mass spectrometry (GC/MS) data of tea extracted by stir bar sorptive extraction.

## Materials and Methods

### Sample Collection

Tea leaves were collected from four communities located at high and low elevations in two major tea-producing provinces of China during the spring and summer harvest seasons from 2014–2016. This includes one high (1651 m) and one low (1162 m) elevation tea-producing community in Menghai County in Yunnan Province, which grow the large-leaf variety of tea (*Camellia sinensis* var. *assamica*). The spring sampling occurred in March, with summer sampling in June. The other tea-producing communities are in Anxi County in Fujian Province and included one high (690 m) and one low (112 m) elevation site, which grow the small-leaf variety (*Camellia sinensis* var. *sinensis*). The spring sampling occurred in May, with the summer sampling in July. Samples consisted of the terminal bud plus two adjacent leaves from five plants in three plots within three tea farms in each community, collected over three consecutive days. Since results of a previous study showed no significant plot-to-plot differences on the same farm (Ahmed et al., 2014), samples from each plot were pooled and homogenized to produce daily samples (n = 3) and treated as independent replicates within each sampling period at each site. A microwave oven was used in the field to stop enzymatic oxidation (Ahmed et al., 2014; Kowalsick et al., 2014). The dried leaves were shipped to Tufts University, where they were stored at −20°C until analyzed.

### Sample Preparation

Aqueous infusions were prepared by brewing 3 g of tea in 30 ml of deionized water at 90°C and cooling to room temperature for 30 min in a closed container. 10ml aliquots were syringe filtered (0.45-µm polytetrafluoroethylene, Fisher Scientific, Pittsburgh, PA) into 10ml Teflon-sealed vials. Organics sorbed into a 0.5-mm thick × 10-mm long polydimethylsiloxane stir bar (Gerstel, Mülheiman der Ruhr, Germany) stirred at 1,200 rpm for 1 h. Stir bars were removed from the vials, rinsed with deionized water, dried with a lint-free wipe, and placed into glass desorption tubes for analysis.

### GC/MS Analysis

Analyses were performed on an Agilent (Santa Clara, CA) 6890/5975 GC/MS equipped with a MultiPurpose Sampler (Gerstel). The thermal desorption unit (TDU, Gerstel) provided splitless transfer of the sample from the stir bar into a programmable temperature vaporization inlet (CIS, Gerstel). The TDU was heated from 40°C (0.70 min) to 275°C (3 min) at 600°C/min under 50 ml/min of helium. After 0.1 min, the CIS, operating in solvent vent mode, was heated from −100°C to 275°C (5 min) at 12°C/s. The GC column (30 m × 250 µm × 0.25 µm RXI-5MS, Restek, Bellefonte, PA) was heated from 40°C (1 min) to 280°C at 5°C/min with 1.2 ml/min of constant helium flow. The MS operated in full scan mode between 40 and 350 m/z, with an electron impact ionization energy of 70 eV. The ion source and quadrupole temperatures were 230°C and 150°C, respectively. A standard mixture of C_7_-C_30_ *n*-alkanes (Sigma-Aldrich, St. Louis, MO) was used to calculate the retention index of each compound. Concentration differences were calculated as relative peak area differences for each compound using naphthalene-d^8^ (Restek) as an internal standard. A total of 300 reference standards were purchased from Sigma-Aldrich, Fisher Scientific, Alfa Aesar (Ward Hill, MA), TCI (Tokyo, Japan), Acros Organics (Pittsburgh, PA) and MP Biomedicals (Santa Ana, CA) to confirm compound identity.

### Data Analysis

Ion Analytics (Gerstel) data analysis software was used to analyze the samples based on a target/nontarget approach as previously described in (Robbat Jr et al., 2017; Kfoury et al., 2018a).

### Statistical Analysis

Principal component analysis (PCA) and partial least squares-discriminant analysis (PLS-DA) were performed on autoscaled (mean-centered and unit-variance scaled) data using MetaboAnalyst 4.0 (Chong et al., 2018). Permutational multivariate analysis of variance (PERMANOVA) was conducted using 999 permutations using the *vegan* package in R (R, 2014; Oksanen et al., 2018). PCA was used to visualize group differences with confirmation made by PERMANOVA. Because current implementations of PLS-DA cannot account for factorial experimental designs, PLS-DA was used only to identify important metabolites for differentiating levels of one variable (e.g., elevation) across all levels of other variables (e.g., across all years and seasons). The quality of the PLS-DA model is described by R^2^ and Q^2^ values (Eriksson et al., 2006). R^2^ measures the degree of fit of data to the model. A seven-fold cross validation was used to produce Q^2^, which measures the predictive power of the model. A feature of PLS-DA is the ability to summarize predictor variable importance across all predictive components with a variable importance in projection (VIP) score (Chong and Jun, 2005). Metabolites with a VIP > 1.0 and statistically different among levels of a response variable (Mann-Whitney test, p < 0.05) were considered important discriminators. Metabolite diversity was calculated as a Simpson’s diversity index (1-D) and the effects of season, elevation year, and their interactions on metabolite diversity were assessed using an ANOVA (p < 0.05).

### Climate Data

Daily maximum and minimum temperatures were obtained from the Climate Prediction Center’s Global Daily Temperature dataset with each grid cell spanning 0.5° latitude × 0.5° longitude (CPC, 2018). Daily precipitation totals were obtained from the Integrated Multi-satellitE Retrievals (IMERG) for Global Precipitation Measurement (GPM) dataset (Huffman et al., 2014), with a spatial resolution of 0.1° latitude × 0.1° longitude. Values are derived from passive microwave sensors from the GPM constellation and ground-based rain gauge observations. Since the IMERG data were not available prior to March 12, 2014, some spring 2014 days are not included in the analysis. The coordinates for each site and corresponding data were imported into a Geographic Information System. Temperature and precipitation values at each site were extracted from corresponding grid cells.

## Results

**Figure 1:**
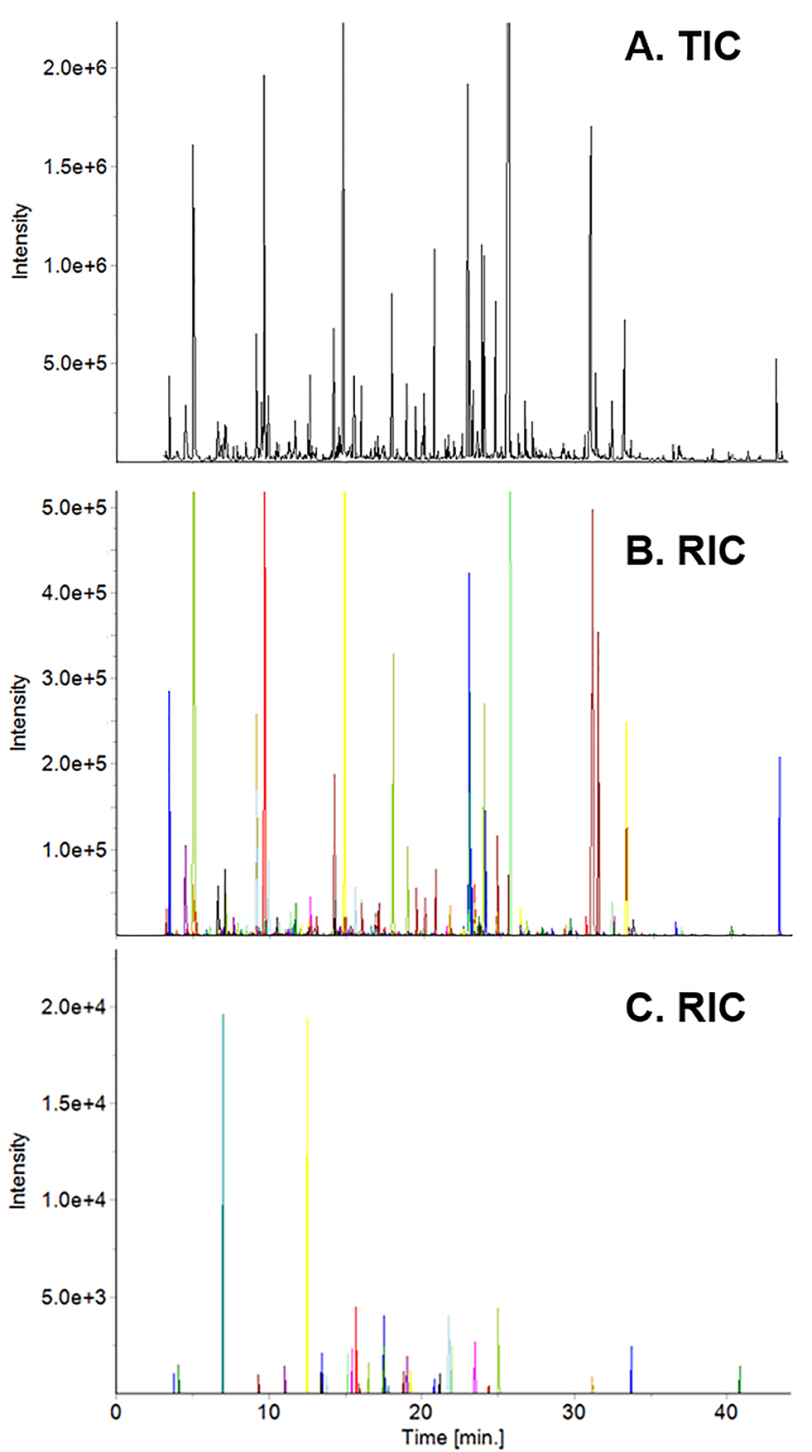
Total ion current (TIC) chromatogram of high elevation spring tea from Fujian **(A)** and reconstructed ion current (RIC) chromatograms of target **(B)** and non-target **(C)** compounds. Each colored peak in the RIC corresponds to a specific compound in the sample.

Yunnan teas, including the ones used in this study, were used to create a Yunnan-specific database of ∼600 compounds. The database served as the initial list of target compounds when we analyzed tea from Fujian based on our targeted/untargeted workflow. For example, spectral deconvolution of the total ion current chromatogram (TIC, **Figure 1A**) of high elevation, spring tea yielded 444 target compounds (**Figure 1B**), where each colored peak indicates a single compound. Subtraction of the mass spectra for these target compounds revealed 32 compounds (nontargets) specific to this sample (**Figure 1C**). By adding these compounds to the database, they become target compounds for the next sample. Subtraction of their mass spectra in subsequent samples reveals new metabolites that are specific to that sample. Based on this approach, we detected 518 volatile metabolites, 58 of them unique to plants grown in Fujian from 2014–2016. Similarly, 506 metabolites were detected, 46 unique, in plants grown in Yunnan over the same time. It should be noted that none of the compounds detected, including unknowns, are known pesticides in the NIST and Wiley databases.

**Figure 2:**
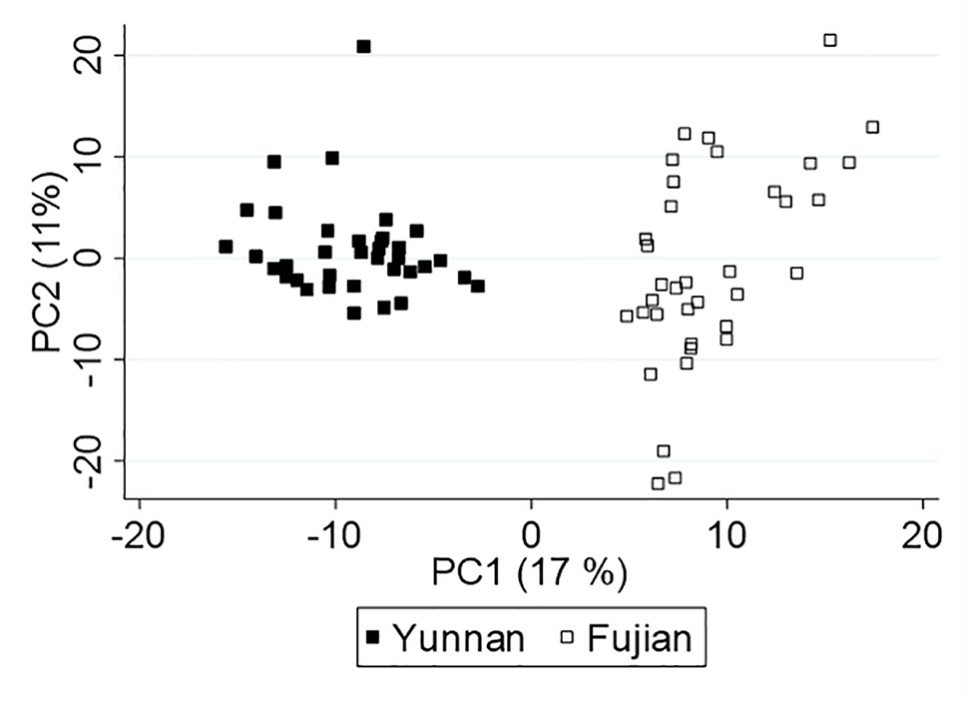
Principal component analysis (PCA) score plot of Yunnan and Fujian tea.

Although Yunnan and Fujian plants produced 460 common compounds (∼90%), differences in their concentration as well as unique metabolites yielded a PCA score plot, **Figure 2**, that separated the samples by location (and/or variety), which we expected due to differences in terroir and farmer practices. Interestingly, the monoterpenes are negatively correlated with PC1 and, therefore, more concentrated in Yunnan tea. Whereas, the sesquiterpenes correlate positively with PC1 and are higher in concentration in Fujian tea. Because of the inherent differences in tea leaf chemistry, we analyzed the data from each province separately to understand the effect of climate on plant metabolites.

### Plant-Climate Interactions: Yunnan Tea

**Table 1** lists the 10-day cumulative rainfall and average temperature prior to each harvest from 2014–2016. We selected this time period based on previous studies, where differences in metabolite chemistry were observed 5 days after the onset of the East Asian Monsoon rains (Ahmed et al., 2014; Kowalsick et al., 2014). Spring rainfall was ≤ 0.1 mm and summer 58 ± 14 mm, with the seasonal difference in temperature ∼3.5 ± 1°C. Although the two elevational sites, from which the samples were harvested, fall within the same latitude/longitude grid cell for temperature, we estimated a 3°C difference between the high (cooler) and low (warmer) elevations using the adiabatic lapse rate (NOAA, 2017).

**Table 1:** 10-day cumulative rainfall and average temperature prior to each harvest (Huffman et al., 2014; CPC, 2018).

Yunnan	Elevation (m)	10-day period	Rain (mm)	Temp (°C)	Fujian	Elevation (m)	10-day period	Rain (mm)	Temp (°C)
2014	1651	March 8–17	0.0	22.9±0.8	2014	690	May 1–10	113.2	18.7 ± 2.4
		May 31–June 9	72.3	28.1±1.1			July 21–30	92.3	26.6 ± 1.4
	1162	March 6–15	0.0	22.4 ± 0.7		112	April 21–30	36.0	20.8 ± 2.0
		May 29 – June 7	40.1	28.5 ± 0.9			July 18–27	100.2	28.6 ± 1.8
2015	1651	March 7–16	0.0	23.7 ± 0.4	2015	690	May 1–10	89.1	21.4 ± 2.1
		June 6–15	69.2	26.7 ± 1.0			July 20–29	156.6	24.8 ± 1.4
	1162	March 5–14	0.0	23.6 ± 0.4		112	April 21–30	43.3	21.9 ± 2.4
		June 4–13	69.4	27.2 ± 1.0			July 17–26	140.6	26.7 ± 1.4
2016	1651	March 12–21	0.1	24.7 ± 0.7	2016	690	April 25–May 4	64.4	21.5 ± 2.0
		June 12–21	48.3	25.8 ± 0.9			July 17–26	9.3	27.2 ± 1.8
	1162	March 10–19	0.1	24.4 ± 0.7		112	April 21–30	68.6	22.2 ± 2.1
		June 10–19	47.3	25.8 ± 0.9			July 15–24	10.6	29.0 ± 1.1

Rainfall data prior to March 12, 2014 was not available and could not be included in the analysis.

**Figure 3:**
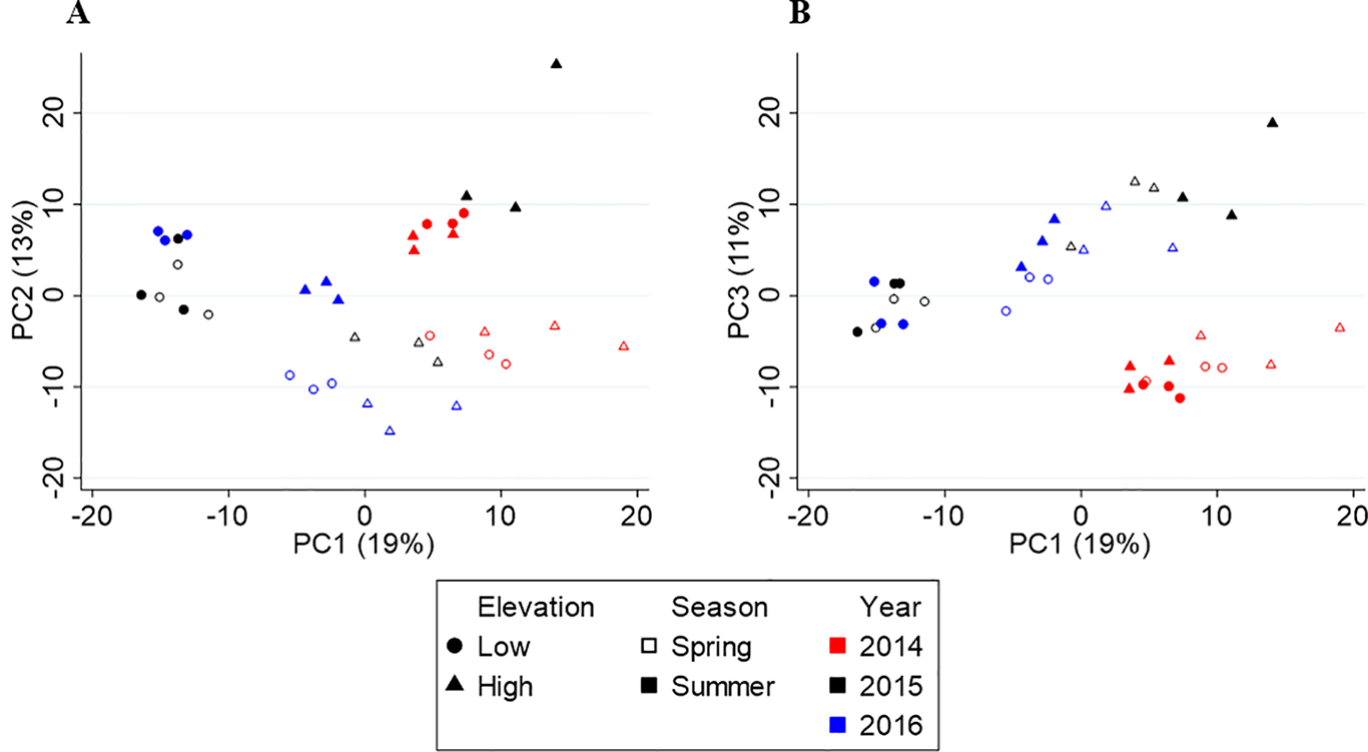
Principal component analysis (PCA) score plots of Yunnan tea. **(A)** PC1 vs. PC2. **(B)** PC1 vs. PC3.

**Figure 3** shows the PCA score plots of PC1 vs. PC2 (A) and PC1 vs. PC3 (B), which explain 43% of the variation in metabolite chemistry. PCA separated the metabolite profiles by elevation (circles vs. triangles) on PC1 (except for 2014), season (open vs. closed shapes) on PC2, and year, 2014 (red) from 2015/2016 (black/blue), on PC3. The separations were confirmed by three-way PERMANOVA showing a significant main effect of elevation (F_(1,24)_ = 34.57, p = 0.001), season (F_(1,24)_ = 11.23, p = 0.001), and year (F_(2,24)_ = 13.05, p = 0.001). PERMANOVA also revealed significant interactive effects between year and elevation (F_(2,24)_ = 13.29, p = 0.001) and year and season (F_(2,24)_ = 4.01, p = 0.010), but not season and elevation (F_(2,24)_ = 0.78, p = 0.425). These interactive effects indicate that there is a different elevational and seasonal effect in at least one of the 3 years. As seen in the score plot (**Figure 3A**), 2014 samples do not separate by elevation along PC1, unlike 2015 and 2016 samples. In addition, 2015 samples separate differently by season than 2014/2016 samples along PC2.

Metabolite diversity was described by the Simpson’s diversity index (Simpson, 1949), which accounts for the number of compounds and their relative abundances in the samples. An index value of 0 means complete dominance of one compound and 1, complete evenness of all compounds. As the number and evenness of compounds increases, namely more compounds with similar relative peak areas, so does the diversity index. Metabolite diversity index ranged from 0.81 to 0.95 and was significantly affected by elevation (F_(1,24)_ = 10.85, p = 0.003), season (F_(1,24)_ = 30.42, p < 0.001), and year (F_(2,24)_ = 19.60, p < 0.001). There were significant interactions between season and year (F_(2,24)_ = 7.42, p = 0.003) and between elevation and year (F_(2,24)_ = 4.94, p = 0.016), but not between season and elevation (F_(2,24)_ = 0.27, p = 0.609). These results match the PERMANOVA for the metabolite relative peak area data. In general, metabolite diversity was greatest in low elevation sites and summer, meaning these plants produced a greater number of compounds having similar metabolite concentrations in response to the conditions studied.

We used PLS-DA to identify metabolites that largely vary between elevations, seasons, and years, independently. The models for elevation (R^2^ = 0.867, Q^2^ = 0.659), season (R^2^ = 0.884, Q^2^ = 0.736), and year (R^2^ = 0.854, Q^2^ = 0.782) had high explanatory and predictive power. We identified 138 metabolites that discriminated high from low elevation (**Supplementary Table S1**), 129 metabolites that differentiated spring from summer (**Supplementary Table S2**), and 155 metabolites that separated years (**Supplementary Table S3**), where the main separation effect is between 2014 and 2015/2016. Some metabolites are affected by more than one climate variable resulting in 353 discriminator compounds.

### Plant-Climate Interactions: Fujian Tea

**Table 1** also lists the climate data for Fujian Province. In contrast to Yunnan, a larger temperature increase occurs from spring to summer (∼6.1 ± 1°C), but rainfall patterns were erratic from year to year. The high and low elevation sites are ∼32 km apart from each other and can therefore be distinguished by latitude/longitude climate grids.

**Figure 4:**
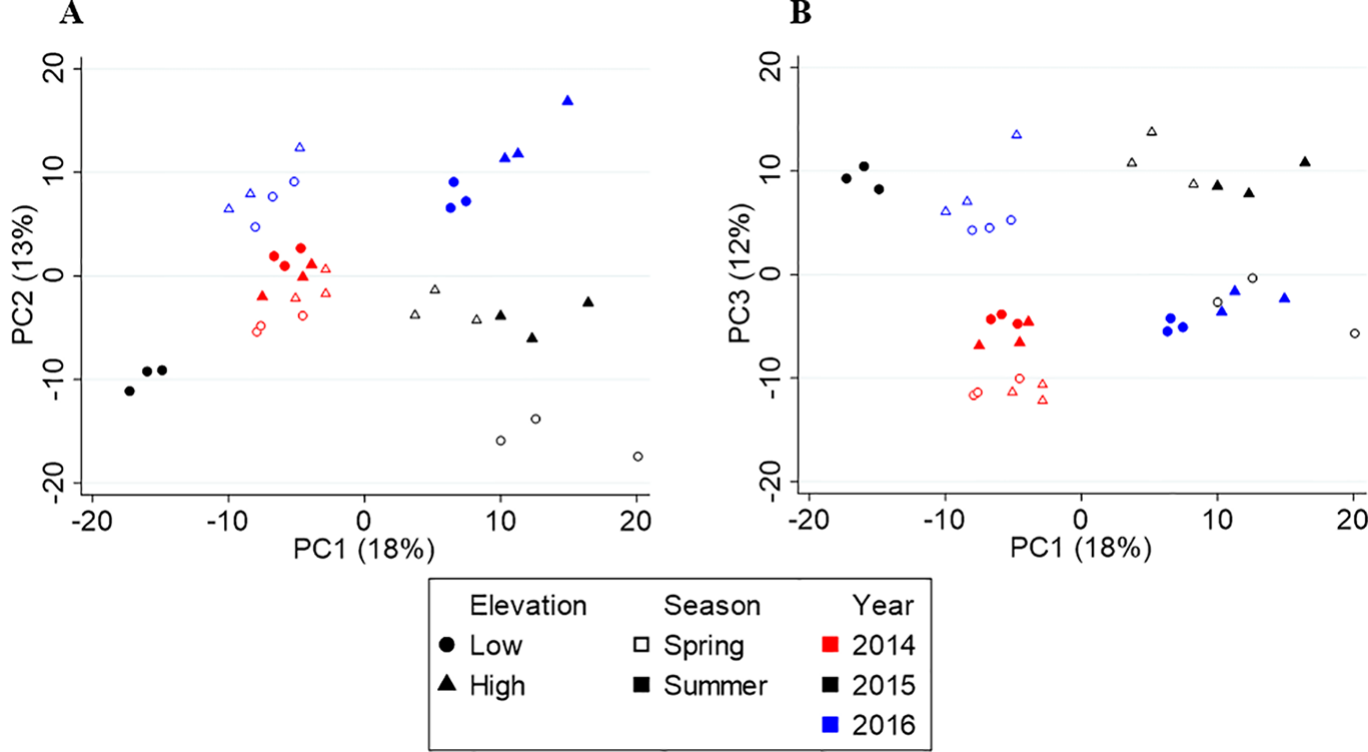
Principal component analysis (PCA) score plots of Fujian tea. **(A)** PC1 vs. PC2. **(B)** PC1 vs. PC3.

**Figure 4** shows the score plots of PC1 vs. PC2 (A) and PC1 vs. PC3 (B). Like Yunnan, the first three axes explain 43% of the variation in the data. The score plot revealed metabolite profiles separated by year (red, black, and blue) on PC2, but no clear seasonal or elevational separation was found. The three-way PERMANOVA confirmed the yearly separation (F_(2,24)_ = 7.61, p = 0.001) and lack of elevational separation (F_(1,24)_ = 1.96, p = 0.134), but revealed a significant seasonal separation (F_(1,24)_ = 3.79, p = 0.024). Upon further inspection, a seasonal separation (open vs. closed shapes) along PC4 was found (**Supplementary Figure S1**). There was also a significant interaction between year and season (F = 4.345, df = 2, p = 0.004) but not year and elevation (F = 1.686, df = 2, p = 0.144) or season and elevation (F = 1.470, df = 1, p = 0.205).

Metabolite diversity in Fujian varied significantly by year (F_(2,24)_ = 5.10, p = 0.014) and season (F_(1,24)_ = 4.32, p = 0.049), but not elevation (F_(1,24)_ = 0.003, p = 0.953). None of the two-way or three-way interactions were significant (all p > 0.1). Metabolite diversity was highest in 2014, which had the largest temperature difference between spring and summer (∼8°C), and higher in summer compared to spring.

PLS-DA was used to identify metabolites that largely vary between seasons and years, independently. The models for season (R^2^ = 0.913, Q^2^ = 0.711) and year (R^2^ = 0.901, Q^2^ = 0.817) had high explanatory and predictive power. We identified 101 metabolites that differentiated spring from summer (**Supplementary Table S4**), and 133 metabolites that separated years (**Supplementary Table S5**). Of the 518 metabolites detected in Fujian teas, 226 discriminate the seasonal and/or yearly differences.

## Discussion

### Elevational Variation

While the elevation difference (∼500 m) is the same in the two provinces, it was the largest source of metabolite variation in Yunnan yet had no effect in Fujian. The Yunnan sites are ∼1,000 m higher in elevation, meaning the Fujian sites are ∼6°C warmer than even the low elevation site in Yunnan (NOAA, 2017). The much warmer temperatures at the Fujian sites could account for the lack of elevational variation seen. In addition, while we classified the site located at 690 m in Fujian as high elevation based on being 500 m higher than the other Fujian site and the elevations found in the area, an elevation of 690 m is not generally regarded as high elevation within the tea industry for high-quality tea.

In Yunnan, four compounds (43, 165, 168, 193—all unknowns) were unique to high elevation samples and four compounds (75, verbenone, 4-methyldecane, 4-ethylbenzaldehyde) were unique to low elevation samples. These compounds could be important biomarkers for distinguishing high and low elevation teas and if the unknowns prove to be of sensory or health importance, identification is necessary. In addition, high elevation teas contain greater concentrations of compounds that have been described as sweet, floral, and fruity (Perflavory, 2015), such as *Z*-jasmone, maltol, methyl 4-methylbenzoate, and bergamal. In contrast, the low elevation teas contain higher concentrations of green, earthy, waxy, and camphoraceous compounds such as 2-phenyl-2-propanol, nonanal, dodecanal, and verbenone (Perflavory, 2015). These results correspond with farmer perceptions that high elevation teas are generally of higher quality due to the sweet, floral, honey-like, and fruity aromatics (Owuor et al., 1990; Ahmed and Stepp, 2012; Han et al., 2017).

In contrast to previous work on Yunnan tea where only discriminating compounds with higher concentrations in high elevation tea had reported health benefits (Kfoury et al., 2018b), in this study compounds with positive correlations to both high and low elevation teas have reported health-beneficial properties (**Supplementary Table S1**). Nutraceutical compounds that distinguish high from low elevation tea include (*E*)-caryophyllene (anticancer, antidepressant, anti-inflammatory), isoeugenol (antibacterial, antioxidant), *epi*-α-cadinol (antibacterial, anti-inflammatory), (*E*)-nerolidol (antianxiety, antimalarial, anticancer), and α-pinene (antiviral, analgesic) (Astani et al., 2010; Tung et al., 2010; Russo, 2011; Guimarães et al., 2013; Bahi et al., 2014; Fidyt et al., 2016; Guerrini et al., 2016; Marteau et al., 2016; Shan et al., 2016; Resende et al., 2017). Health beneficial compounds that differentiate low from how elevation tea exhibit antibacterial (verbenone, decanal, undecanal, dodecanal), antifungal (nonanal), and antiseptic (2-phenoxyethanol) properties (Buhrer et al., 2002; Battinelli et al., 2006; Fujita et al., 2015; Iscan, 2017). Although the volatile metabolites represent a small fraction of the total tea mass, findings indicate that volatile tea extracts have proven health benefits (Takahashi et al., 2014; Li et al., 2017). However, further studies are needed to determine if the compounds found here are present in adequate concentrations to provide these purported health benefits.

### Seasonal Variation

Although rainfall and temperature generally increase from spring to summer in both Yunnan and Fujian, Yunnan experiences a striking increase in rain and smaller changes in temperature compared to Fujian, which has a larger temperature increase and erratic rainfall from year to year. The leaves from both provinces had significant chemical variations due to seasonal changes. Interestingly, nine compounds (N-ethylsuccinimide, isomenthone, (*E*)-β-ocimene, menthone, 160, flouranthene, 1-ethyl-1H-pyrrole, 76, 1-ethyl-1H-pyrrole-2-carboxaldehyde) were discriminators of spring tea and five compounds (36, 167, 181, 224, 2-phenoxyethanol) discriminate summer tea in both Yunnan and Fujian (**Supplementary Tables S2** and **S4**). In addition, 1-ethyl-1H-pyrrole was unique to spring tea in both locations and could be a key spring tea biomarker, independent of location, and variety.

Regardless of the inherent differences between the provinces, more compounds that have been described as sweet, floral, honey-like, and fruity are discriminators of spring tea (Perflavory, 2015). These include (*E*)-β-ocimene, 2,2,6-trimethylcyclohexanone, (*Z*)-jasmone, phenylethyl acetate, and 5-methylfurfural. On the other hand, 1-octen-3-ol, (2*E*,4*E*)-heptadienal, 2-pentylfuran, α-copaene, and *cis*-calamenene are higher in concentration in summer tea and are characterized as green, herbal, earthy, and woody (**Supplementary Tables S2** and **S4**). In addition, compounds significant to both seasons have reported health benefits (**Supplementary Tables S2** and **S4**). Spring-associated compounds include menthone (antibacterial, anti-inflammatory), eucalyptol (antibacterial, cardioprotective), indole (antibacterial, antifungal), and coumarin (anti-inflammatory, anticancer) (Egan et al., 1990; Muroi and Kubo, 1993; Nidiry and Babu, 2005; Santos et al., 2011; Shan et al., 2016; Iscan, 2017). Summer-associated compounds include (*E*)-β-ionone (anticancer, antibacterial), borneol (anti-inflammatory, analgesic), methyl anthranilate (antifungal), and quinoline (antimalarial, anticonvulsant, anticancer) (Muroi and Kubo, 1993; Nidiry and Babu, 2005; Oz et al., 2015; Anjali and Singh, 2016; Furtado Kelly et al., 2016).

Interestingly, in Yunnan, many of the discriminating metabolites are in both spring and high elevation or in the summer and low elevation teas. For example, spring and high elevation teas contain significantly higher amounts of 2-hydroxy-5-methylacetophenone, isoeugenol, 4-methylbenzaldehyde, and norfuraneol that are described as sweet, fruity, floral, honey-like compounds (Perflavory, 2015). In contrast, summer and low elevation teas have significantly higher amounts of 2,6-dimethyl-3,7-octadiene-2,6-diol, 2-phenoxyethanol, octanal, and nonanal that are described as herbal, green, fatty, and metallic (Perflavory, 2015). These findings agree with farmers’ perceptions that high elevation and spring teas are higher in aromatic quality possessing sweet, fruity, floral, honey-like characteristics (Ahmed and Stepp, 2012; Ahmed et al., 2014; Han et al., 2017). Most likely, these metabolites are induced by temperature since spring/high elevations are cooler than summer/low elevations.

### Yearly Variation and Interactive Effects

In Yunnan, both PCA and PLS-DA show the yearly separation is between 2014 and 2015/2016. Based on the climate data, 2014 experienced the coolest spring and warmest summer temperatures compared to 2015 and 2016. Interestingly, many of the higher concentration compounds in the 2014 tea were also higher in concentration in either spring or summer tea. For example, compounds such as (*E*)-β-ocimene, (*Z*)-herboxide, (3*Z*)-hexenyl acetate, and safranal were higher in concentration in 2014 and spring teas and are described as sweet, herbal, fruity aromas (Perflavory, 2015). Compounds such as (4*Z*)-heptenal, pyridine, and 2-methyldecane were higher in concentration in 2014 and summer teas and are characterized as fatty, green, and fishy (Perflavory, 2015). These compounds are likely being induced by the cooler spring or warmer summer temperatures. In contrast, compounds higher in concentration in 2015/2016 are not affected by seasonal variations such as 4-ethyl-2-methoxyphenol (smoky, phenolic), catechol (no aroma), 4-methyl-3-penten-2-one (sweet, earthy), and γ-heptalactone (sweet, nutty) (Perflavory, 2015), which are likely not influenced by temperature changes. Another indication that 2014 differed from 2015/2016 is that 2014 samples do not separate by elevation, which is confirmed by the interactive effect between year and elevation. Compounds such as hexanoic acid, 2-nonanone, decanal, (*E*)-herboxide, 4-methylbenzaldehyde, and 2-methoxy-4-vinylphenol exhibit no change in concentration between elevations in 2014, but are significantly higher in concentration at one elevation or the other in 2015/2016 tea.

Also, 2014 and 2016 samples separate similarly by season whereas 2015 samples do not follow the same pattern along PC2, which is confirmed by the interactive effect between year and season. While several compounds exhibit the opposite change in concentration from spring to summer, others show a more or less enhanced concentration change in 2015 compared to 2014/2016. For example, (*E*)-β-ocimene, methyl salicylate, and 7-methoxycoumarin exhibit opposite concentration changes from spring to summer in 2015 compared to 2014/2016. In addition, hexanol, isomenthone, and camphor have a greater concentration difference between spring and summer whereas 2*E*-hexenal, benzenacetonitrile and 4-methyl-3-penten-2-one have a smaller difference in 2015. The climate data do not provide any indication as to what is causing these interactive effects. However, (*E*)-β-ocimene, methyl salicylate, 2*E*-hexenal, and 4-methyl-3-penten-2-one are induced by herbivory in tea, so these year-to-year differences could reflect differences in the timing of pest outbreaks (Cai et al., 2014; Kfoury et al., 2017; Scott et al., 2019).

Year-to-year variations account for the greatest source of variation in Fujian and is likely due to the inconsistent rainfall patterns seen from year-to-year (**Table 1**). The significant interactive effect between year and season confirms this finding, not found between year and elevation or season and elevation. In 2016, the plants experienced an extremely dry summer, which is the reverse of the previous two years. As a result, many metabolite concentrations increased/decreased in the opposite direction from 2014 and 2015. For example, safranal (sweet, herbal), norfuraneol (sweet, caramel), cyclohexanone (minty), o-xylene (geranium), isoeugenol (floral, clove), and geranyl acetone (floral, fruity) are higher in concentration in spring tea in 2014 and 2015, but higher in summer tea in 2016. Similarly, 2-ethylhexanol (green, oily), camphor (camphor, medicinal), methyl hexanoate (fruity, fatty), biphenyl (floral, green), and 5-ethyl-2(5H)-furanone (no aroma) are higher in concentration in 2014/2015 summer, compared to the 2016 spring tea. It is likely that the changes in concentration of these and many other metabolites that behave similarly are the result of changes in rainfall.

## Conclusion

In this work, we demonstrated that our target/nontarget approach provides efficient and comprehensive analysis of a complex, natural product, namely, tea. Findings show the differences in season, elevation and year cause significant and interactive alterations in tea chemistry. Independent of location, cooler temperatures in the spring and at high elevation concomitant with lower rainfall, results in higher concentrations of compounds with aromas characteristic consistent with farmers’ perceptions of high-quality tea. Although many of the metabolites identified have reported health-beneficial properties studies are needed to assess efficacy and identify unknowns that are discriminator compounds that might also contribute to quality. Given the interactive effects found between years and season/elevation, future studies should be cautious in drawing conclusions based on only 1 year of sampling and more multi-year studies should be conducted to assist farmers with the challenge of producing a consistently high quality product. More natural-human systems investigations are needed to assess plant response under ever changing environmental conditions.

## Data Availability Statement

The datasets generated for this study are available on request to the corresponding author.

## Author Contributions

AR, CO, SA, SC, TG, and JS acquired funding and conceptualized the project. DX, CL, SA, and WH supported with in-field sample collections and logistics. CM acquired and analyzed the climate data. NK acquired and analyzed the metabolite data. NK and ES performed statistical analysis. NK, AR, ES, and CO wrote the manuscript draft. All authors contributed to manuscript revision and approved the submitted version.

## Funding

This work was supported by the National Science Foundation, Grant BCS-1313775.

## Conflict of Interest

AR is the founder of Ion Analytics.

The remaining authors declare that the research was conducted in the absence of any commercial or financial relationships that could be construed as a potential conflict of interest.
