# Supplementary material for: Plant-Climate Interaction Effects: Changes in the Relative Distribution and Concentration of the Volatile Tea Leaf Metabolome in 2014–2016
Source: Front Plant Sci. 2019 Nov 22;10:1518. doi: 10.3389/fpls.2019.01518 (PMC6882950; doi:10.3389/fpls.2019.01518)
Supplement: Supplementary file 1 [file DataSheet_1.docx]

Supplementary Material

**Table S1.** Statistically important metabolites in high and low elevation Yunnan tea

| **Compound** | **VIP** | **p-value** | **RPA**  **High** | **RPA**  **Low** | **Aroma^a^** | **Health Property** |
| --- | --- | --- | --- | --- | --- | --- |
| **High** | | | | | | |
| 165 | 2.83 | 0.0001 | 0.0004 | - |  |  |
| 222 | 2.67 | 0.0001 | 0.0006 | 0.0001 |  |  |
| 52 | 2.37 | 0.0003 | 0.0015 | 0.0003 |  |  |
| 7-Methoxycoumarin | 2.24 | 0.0001 | 0.0016 | 0.0001 | sweet, balsamic | antinociceptive^1^ anticancer^2^  anti-inflammatory^3^ |
| 43 | 2.12 | 0.0006 | 0.0007 | - |  |  |
| Pyrethrone | 2.12 | 0.0001 | 0.0016 | 0.0009 |  |  |
| p-Xylene | 2.10 | 0.0001 | 0.0063 | 0.0042 | sweet, grain^4^ |  |
| (3*Z*)-Hexenyl isovalerate* | 2.08 | 0.0002 | 0.0003 | 0.0001 | green, fruity |  |
| 154 | 2.01 | 0.0001 | 0.0028 | 0.0010 |  |  |
| 2,3-Dimethylhexane* | 2.01 | 0.0001 | 0.0016 | 0.0009 |  |  |
| 2,4-Di-tert-butylphenol | 1.97 | 0.0002 | 0.0298 | 0.0238 |  | antioxidant^5^ |
| Lavender lactone | 1.93 | 0.0003 | 0.0006 | 0.0004 | fruity, minty |  |
| 168 | 1.90 | 0.002 | 0.0003 | - |  |  |
| Butyl p-toluate | 1.89 | 0.0002 | 0.0015 | 0.0010 |  |  |
| Methyl o-anisate | 1.88 | 0.0001 | 0.0011 | 0.0003 | floral, fruity |  |
| 4-Methylbenzaldehyde* | 1.85 | 0.0006 | 0.0043 | 0.0028 | fruity, cherry | antiviral^6^ |
| Geranic acid | 1.82 | 0.0008 | 0.0056 | 0.0030 | green, woody |  |
| 173 | 1.81 | 0.0004 | 0.0054 | 0.0035 |  |  |
| 55* | 1.79 | 0.001 | 0.0015 | 0.0012 |  |  |
| 97 | 1.79 | 0.002 | 0.0018 | 0.0013 |  |  |
| (*Z*)-Jasmone | 1.78 | 0.0002 | 0.0120 | 0.0042 | floral, jasmine | antibacterial^7^ anticancer^8^ |
| 172* | 1.77 | 0.002 | 0.0028 | 0.0020 |  |  |
| 4* | 1.77 | 0.002 | 0.0025 | 0.0016 |  |  |
| Toluene | 1.76 | 0.002 | 0.3198 | 0.1235 | sweet, paint |  |
| 128* | 1.76 | 0.002 | 0.0005 | 0.0003 |  |  |
| (*E*)-Caryophyllene | 1.75 | 0.003 | 0.0003 | 0.0002 | sweet, clove, woody | anticancer^9^ antibacterial^7^ antianxiety antidepressant^10^  anti-inflammatory^11^ |
| 35* | 1.74 | 0.001 | 0.0182 | 0.0126 |  |  |
| 2,2,4-Trimethylhexane* | 1.74 | 0.0006 | 0.0015 | 0.0010 |  |  |
| 4,6-Dimethyl-2-heptanone | 1.73 | 0.001 | 0.0040 | 0.0031 | fruity |  |
| 47 | 1.69 | 0.0009 | 0.0007 | 0.0004 |  |  |
| 118* | 1.68 | 0.001 | 0.0016 | 0.0011 |  |  |
| 6-Methyl-3,5-heptadiene-2-one | 1.68 | 0.0001 | 0.0043 | 0.0007 | sweet, coconut |  |
| 2,4-Dimethyl-1-heptene | 1.68 | 0.004 | 0.0016 | 0.0010 |  |  |
| 186 | 1.67 | 0.0001 | 0.0018 | 0.0013 |  |  |
| Styrene | 1.65 | 0.003 | 0.0438 | 0.0330 | sweet, floral, balsamic |  |
| 56* | 1.64 | 0.005 | 0.0012 | 0.0008 |  |  |
| Bergamal | 1.64 | 0.001 | 0.0008 | 0.0005 | floral, fruity, earthy |  |
| α-Muurolene* | 1.62 | 0.006 | 0.0004 | 0.0003 |  |  |
| 4-Methyl-2-heptanone* | 1.61 | 0.01 | 0.0003 | 0.0002 |  |  |
| 204* | 1.60 | 0.002 | 0.0090 | 0.0066 |  |  |
| 80* | 1.59 | 0.01 | 0.0012 | 0.0007 |  |  |
| *epi*-α-Murrolol* | 1.58 | 0.001 | 0.0055 | 0.0030 | herbal, spicy | antibacterial^12^ antioxidant^13^ |
| Octane | 1.57 | 0.009 | 0.0119 | 0.0084 | gasoline |  |
| 61* | 1.57 | 0.004 | 0.0011 | 0.0008 |  |  |
| Isoeugenol* | 1.57 | 0.004 | 0.0022 | 0.0012 | floral, clove, woody | anitbacterial^14^ antioxidant^15^ |
| 193 | 1.56 | 0.0001 | 0.0008 | - |  |  |
| (2*E*)-Undecenal | 1.56 | 0.005 | 0.0019 | 0.0014 | fruity, green | antileishmanial^16^ |
| 9-Hexadecenoic acid | 1.55 | 0.006 | 0.0027 | 0.0017 |  | anti-inflammatory^17^ |
| β-Calacorene* | 1.55 | 0.003 | 0.0008 | 0.0005 |  |  |
| α-Amorphene* | 1.54 | 0.006 | 0.0005 | 0.0003 |  |  |
| Ethylbenzene | 1.54 | 0.003 | 0.0048 | 0.0037 |  |  |
| Methyl anthranilate | 1.54 | 0.003 | 0.0009 | 0.0005 | fruity, grape | antifungal^18^ |
| 115 | 1.52 | 0.005 | 0.0006 | 0.0005 |  |  |
| 189 | 1.50 | 0.001 | 0.0030 | 0.0012 |  |  |
| 155 | 1.50 | 0.008 | 0.0100 | 0.0075 |  |  |
| 124 | 1.47 | 0.002 | 0.0009 | 0.0002 |  |  |
| Tridecanoic acid* | 1.46 | 0.01 | 0.0016 | 0.0011 | waxy, woody |  |
| 2,4,4-Trimethyl-1-pentene | 1.46 | 0.008 | 0.0026 | 0.0019 |  |  |
| Decane* | 1.45 | 0.004 | 0.0017 | 0.0011 |  |  |
| Maltol | 1.45 | 0.006 | 0.0059 | 0.0039 | sweet, marshmallow | antianxiety^19^ antioxidant^20^ |
| *epi*-α-Cadinol* | 1.44 | 0.003 | 0.0109 | 0.0056 | herbal | antibacterial^12^ anticancer^21^  anti-inflammatory^22^ |
| 95* | 1.44 | 0.02 | 0.0020 | 0.0017 |  |  |
| α-Cadinol* | 1.42 | 0.0009 | 0.0101 | 0.0047 | herbal, woody | antibacterial, antioxidant^12^  anti-inflammatory ^22^ |
| 194 | 1.42 | 0.01 | 0.0004 | 0.0003 |  |  |
| 2-Hydroxy-5-methylacetophenone* | 1.40 | 0.008 | 0.0008 | 0.0006 | sweet, floral, herbal |  |
| 2,6-Dimethyl-2-heptanol | 1.40 | 0.02 | 0.0021 | 0.0015 | floral, woody, herbal |  |
| β-Homocyclocitral* | 1.39 | 0.003 | 0.0012 | 0.0009 | camphor, cooling |  |
| 127* | 1.38 | 0.007 | 0.0007 | 0.0003 |  |  |
| α-Cyclocitral | 1.38 | 0.02 | 0.0005 | 0.0004 |  |  |
| Crotonic acid | 1.38 | 0.0008 | 0.0047 | 0.0005 | milky |  |
| α-Muurolol | 1.37 | 0.001 | 0.0043 | 0.0022 |  | antibacterial, antioxidant^12^ |
| Indole* | 1.36 | 0.03 | 0.0781 | 0.0181 | fecal, mothball, floral | antibacterial^7^ antifungal^18^ |
| 65 | 1.36 | 0.03 | 0.0004 | 0.0003 |  |  |
| Norfuraneol* | 1.35 | 0.02 | 0.0077 | 0.0047 | sweet, caramel |  |
| o-Xylene | 1.35 | 0.009 | 0.0017 | 0.0012 | geranium |  |
| β-Cyclocitral* | 1.34 | 0.01 | 0.0084 | 0.0060 | sweet, herbal, minty |  |
| 1-Ethyl-2-methylbenzene | 1.34 | 0.02 | 0.0003 | 0.0002 |  |  |
| (*E*)-Nerolidol | 1.33 | 0.02 | 0.0027 | 0.0012 | floral, woody | antianxiety, anti-malarial^23^ anticancer^21^ antibacterial^12^  anti-inflammatory^22^ |
| Isoborneol* | 1.33 | 0.009 | 0.0007 | 0.0005 | camphor woody | antiviral, antibacterial^24^ |
| 4,4-Dimethyl-2-pentanone | 1.32 | 0.004 | 0.0030 | 0.0021 |  |  |
| Dodecanamide* | 1.32 | 0.01 | 0.0105 | 0.0070 |  |  |
| Theaspirane B* | 1.32 | 0.02 | 0.0036 | 0.0020 | tea, herbal, honey |  |
| 138* | 1.32 | 0.02 | 0.0018 | 0.0014 |  |  |
| Dimethyl trisulfide* | 1.30 | 0.02 | 0.0006 | 0.0004 | sulfur, cabbage | antioxidant, hepatoprotective^25^ |
| Theaspirane A* | 1.29 | 0.01 | 0.0013 | 0.0008 | tea, herbal, honey |  |
| Methyl pyruvate* | 1.29 | 0.04 | 0.003 | 0.0002 |  |  |
| 219 | 1.29 | 0.02 | 0.0006 | 0.0004 |  |  |
| Cadalene* | 1.27 | 0.006 | 0.0003 | 0.0002 |  | antibacterial, antioxidant^13^ |
| 2,3-Dihydrobenzofuran* | 1.25 | 0.01 | 0.0045 | 0.0029 | green, herbal^26^ |  |
| 1,4-Diacetylbenzene | 1.24 | 0.03 | 0.0013 | 0.0010 |  |  |
| 2-Methoxy-4-vinylphenol* | 1.23 | 0.007 | 0.0025 | 0.0014 | smoky, clove | anti-inflammatory^27^ |
| Pentadecane* | 1.22 | 0.02 | 0.0015 | 0.0012 |  |  |
| 167* | 1.22 | 0.01 | 0.0024 | 0.0017 |  |  |
| *cis*-Calamenene* | 1.19 | 0.009 | 0.0004 | 0.0003 | herbal, spicy | antimalarial^28^ antitumor^29^ |
| Jasmine lactone* | 1.18 | 0.04 | 0.0068 | 0.0031 | jasmine, fruity |  |
| Decanamide | 1.18 | 0.04 | 0.0058 | 0.0042 |  |  |
| Methyl 4-methyl benzoate | 1.17 | 0.03 | 0.0007 | 0.0006 | sweet, anise, floral |  |
| 158* | 1.16 | 0.006 | 0.0010 | 0.0005 |  |  |
| 4-2,4,4-Trimethyl-cyclohexa-1,5-dienyl-but-3-en-2-one* | 1.16 | 0.002 | 0.0018 | 0.0010 |  |  |
| Methyl benzoate* | 1.14 | 0.005 | 0.0034 | 0.0024 | cherry, phenolic |  |
| 4-Methyl-3-penten-2-one* | 1.14 | 0.002 | 0.0149 | 0.0065 | sweet, earthy |  |
| 215 | 1.14 | 0.02 | 0.0022 | 0.0008 |  |  |
| Tetradecanol | 1.14 | 0.03 | 0.0022 | 0.0017 | coconut, fruity, waxy | anti-inflammatory, gastroprotective^30^ |
| α-Pinene | 1.12 | 0.01 | 0.0003 | 0.0002 | sweet, pine, camphor | antibacterial^31^ analgesic^32^ hypotensive^33^ antiviral^34^ |
| (4*Z*)-Heptenal* | 1.12 | 0.03 | 0.0007 | 0.0005 | oily, fatty, green |  |
| 176 | 1.11 | 0.04 | 0.0006 | 0.0005 |  |  |
| 160* | 1.11 | 0.01 | 0.0014 | 0.0007 |  |  |
| (*Z*)-Methyl jasmonate | 1.10 | 0.009 | 0.0093 | 0.0051 | floral, jasmine | anticancer^8^ anti-inflammatory,  antioxidant, neuroprotective, antistress^35^ |
| 218* | 1.08 | 0.04 | 0.0011 | 0.0008 |  |  |
| 202* | 1.06 | 0.03 | 0.0005 | 0.0004 |  |  |
| Muurola-4,1014-dien-1-β-ol* | 1.04 | 0.01 | 0.0018 | 0.0012 |  |  |
| 2-Octanone | 1.02 | 0.04 | 0.0032 | 0.0027 | herbal, earthy |  |
| 26* | 1.01 | 0.002 | 0.0084 | 0.0030 |  |  |
| **Low** | | | | | | |
| Verbenone | 2.55 | 0.0001 | - | 0.0003 | camphor, menthol | antibacterial^31^ |
| 4-Methyldecane | 2.51 | 0.0001 | - | 0.0017 |  |  |
| 75 | 2.15 | 0.0001 | - | 0.0004 |  |  |
| 2-Methyl-1H-pyrrole | 2.09 | 0.0003 | 0.0001 | 0.0003 |  |  |
| 4-Ethylbenzaldehyde | 1.96 | 0.0005 | - | 0.0002 | bitter, almond |  |
| 2-Phenyl-2-propanol | 1.91 | 0.0002 | 0.0005 | 0.0007 | green, sweet, earthy |  |
| Octanal* | 1.73 | 0.001 | 0.0027 | 0.0048 | green, fatty, citrus |  |
| Nonanal | 1.72 | 0.001 | 0.0103 | 0.0193 | cucumber, waxy | antifungal^36^ |
| 90* | 1.70 | 0.01 | 0.0005 | 0.0014 |  |  |
| Decanal | 1.66 | 0.009 | 0.0052 | 0.0140 | orange, green, waxy | antibacterial^37^ |
| 1-Methylpyrrolidinone* | 1.65 | 0.01 | 0.0001 | 0.0007 |  |  |
| 2,6-Dimethyl-3,7-octadiene-2,6-diol* | 1.60 | 0.01 | 0.0003 | 0.0012 | fruity, herbal |  |
| (2*Z*)-Octen-1-ol* | 1.53 | 0.01 | 0.0006 | 0.0011 |  |  |
| Dodecanal | 1.50 | 0.009 | 0.0006 | 0.0008 | citrus, green, waxy | antibacterial^37^ |
| 2-Ethylhexanoic acid* | 1.50 | 0.02 | 0.0001 | 0.0004 |  |  |
| 2-Phenoxyethanol* | 1.49 | 0.03 | 0.0005 | 0.0015 | mild rose, metallic | antiseptic^38^ |
| Isoamyl alcohol | 1.44 | 0.01 | 0.0004 | 0.0016 | alcoholic, banana |  |
| Undecanal | 1.42 | 0.004 | 0.0005 | 0.0011 | citrus, waxy, soapy | antibacterial^37^ |
| 2,4-Dimethylbenzaldehyde* | 1.35 | 0.02 | 0.0005 | 0.0007 | almond, cherry |  |
| Pentyl propanate | 1.32 | 0.03 | 0.0007 | 0.0017 | fruity, apricot |  |
| 94 | 1.32 | 0.04 | 0.0013 | 0.0020 |  |  |
| 57 | 1.21 | 0.005 | 0.0002 | 0.0005 |  |  |
| 87* | 1.17 | 0.02 | 0.0001 | 0.0003 |  |  |
| 2-Phenylphenol | 1.17 | 0.007 | 0.0005 | 0.0008 |  |  |
| 53 | 1.06 | 0.04 | 0.0015 | 0.0019 |  |  |

^a^Aroma information obtained from the Good Scents Company^39^ unless otherwise noted. *Compound is affected by more than one environmental factor. Numbers in the compound column refer to unknown compounds.

**Table S2.** Statistically important metabolites in spring and summer Yunnan tea

| **Compound** | | | **VIP** | **p-value** | **RPA**  **Spring** | **RPA Summer** | **Aroma** | **Health Property** |
| --- | --- | --- | --- | --- | --- | --- | --- | --- |
| **Spring** | | | | | | | | |
| 139 | | | 2.64 | 0.0001 | 0.0013 | 0.0001 |  |  |
| 137 | | | 2.43 | 0.0001 | 0.0016 | 0.0003 |  |  |
| 133 | | | 2.23 | 0.0001 | 0.0012 | 0.0001 |  |  |
| 2-Methylpentanal | | | 2.21 | 0.0001 | 0.0023 | 0.0008 | fruity, green |  |
| 1-Ethyl-1H-pyrrole-2-carboxaldehyde | | | 2.12 | 0.0001 | 0.0149 | 0.0044 | roasted, smoky |  |
| Menthone | | | 2.02 | 0.0006 | 0.0008 | 0.0004 | green, minty | antibacterial^31^ anti-inflammatory^40^ |
| 1-Ethyl-1H-pyrrole | | | 2.00 | 0.0001 | 0.0023 | - | roasted |  |
| Fluoranthene | | | 1.97 | 0.0003 | 0.0029 | 0.0013 |  |  |
| 160* | | | 1.94 | 0.0003 | 0.0016 | 0.0005 |  |  |
| 2-Methoxy-4-vinylphenol* | | | 1.76 | 0.0005 | 0.0027 | 0.0011 | smoky, clove | anti-inflammatory^27^ |
| 2,3-Octanedione | | | 1.74 | 0.002 | 0.0016 | 0.0012 | buttery, broccoli |  |
| 2,3-Dihydrobenzofuran* | | | 1.72 | 0.001 | 0.0049 | 0.0025 | green, herbal^26^ |  |
| 142 | | | 1.70 | 0.004 | 0.0005 | 0.0002 |  |  |
| 4-2,4,4-Trimethyl-cyclohexa-1,5-dienyl-but-3-en-2-one* | | | 1.69 | 0.0004 | 0.0020 | 0.0008 |  |  |
| 180 | | | 1.68 | 0.002 | 0.0008 | 0.0003 |  |  |
| 39 | | | 1.61 | 0.001 | 0.0062 | 0.0033 |  |  |
| 2-Hydroxy-5-methylacetophenone* | | | 1.59 | 0.003 | 0.0008 | 0.0006 | sweet, floral, herbal |  |
| Isoeugenol* | | | 1.58 | 0.004 | 0.0022 | 0.0012 | floral, clove, woody | anitbacterial^14^ antioxidant^15^ |
| 76 | | | 1.57 | 0.0001 | 0.0041 | 0.0010 |  |  |
| Benzyl nitrile* | | | 1.50 | 0.0007 | 0.0049 | 0.0015 | floral^41^ |  |
| (*Z*)-Herboxide* | | | 1.48 | 0.02 | 0.0029 | 0.0015 | herbal, woody |  |
| Safranal* | | | 1.47 | 0.005 | 0.0042 | 0.0025 | sweet, herbal | antinociceptive^42^ antimicrobial^43^ |
| 41 | | | 1.47 | 0.005 | 0.0053 | 0.0038 |  |  |
| Dimethyl trisulfide* | | | 1.46 | 0.006 | 0.0006 | 0.0004 | sulfur, cabbage | antioxidant, hepatoprotective^25^ |
| 204* | | | 1.45 | 0.01 | 0.0090 | 0.0067 |  |  |
| 42 | | | 1.43 | 0.0001 | 0.0008 | - |  |  |
| Benzyl benzoate | | | 1.42 | 0.006 | 0.0064 | 0.0041 | herbal, balsamic | antibacterial^44^ |
| Eucalyptol | | | 1.42 | 0.005 | 0.0004 | 0.0003 | eucalyptus, sweet | antibacterial^31^ analgesic^32^  antiviral^34^ cardioprotective^33^ |
| Phenylethyl acetate | | | 1.42 | 0.0001 | 0.0006 | 0.0001 | rose, honey |  |
| 95* | | | 1.40 | 0.008 | 0.0020 | 0.0017 |  |  |
| Benzylideneacetone | | | 1.39 | 0.01 | 0.0002 | 0.0001 | floral, fruity |  |
| 4-Methyl-2-heptanone* | | | 1.38 | 0.01 | 0.0092 | 0.0066 |  |  |
| 55* | | | 1.37 | 0.01 | 0.0015 | 0.0012 |  |  |
| (3*Z*)-Hexenyl acetate* | | | 1.37 | 0.03 | 0.0025 | 0.0013 | green, sweet, fruity |  |
| 1-Nitro-2-phenylethane | | | 1.36 | 0.001 | 0.0097 | 0.0080 | floral, spice | cardioprotective^45^ |
| Cyclohexanone | | | 1.31 | 0.03 | 0.0005 | 0.0004 | minty |  |
| 118* | | | 1.31 | 0.02 | 0.0015 | 0.0012 |  |  |
| 56* | | | 1.31 | 0.007 | 0.0012 | 0.0009 |  |  |
| 2,3-Dimethylhexane* | | | 1.30 | 0.04 | 0.0015 | 0.0010 |  |  |
| Decane* | | | 1.29 | 0.003 | 0.0053 | 0.0038 |  |  |
| 61* | | | 1.28 | 0.03 | 0.0011 | 0.0008 |  |  |
| Indole* | | | 1.27 | 0.03 | 0.0767 | 0.0196 | fecal, mothball, floral | antibacterial^7^ antifungal^18^ |
| 4-Methylbenzaldehyde* | | | 1.24 | 0.03 | 0.0041 | 0.0030 | fruity, cherry | antiviral^6^ |
| 67 | | | 1.24 | 0.04 | 0.0005 | 0.0004 |  |  |
| Menthol | | | 1.23 | 0.04 | 0.0019 | 0.0012 | peppermint, cooling | antibacterial^31^ decongestant^46^ cardioprotective^33^ analgesic^32^ |
| Isomenthone | | | 1.22 | 0.004 | 0.0002 | 0.0001 | sweet, peppermint |  |
| 110 | | | 1.21 | 0.002 | 0.0173 | 0.0092 |  |  |
| Benzoic acid | | | 1.19 | 0.01 | 0.0036 | 0.0020 | faint balsamic | antibacterial^47^ |
| Norfuraneol* | | | 1.15 | 0.007 | 0.0075 | 0.0049 | sweet, caramel |  |
| (*E*)-Herboxide* | | | 1.13 | 0.04 | 0.0023 | 0.0016 | herbal, woody |  |
| *N*-Ethylsuccinimide | | | 1.13 | 0.04 | 0.0006 | 0.0004 |  |  |
| Ethyl benzoate | | | 1.12 | 0.03 | 0.0034 | 0.0019 | fruity, herbal |  |
| α-Muurolene* | | | 1.11 | 0.02 | 0.0005 | 0.0004 |  |  |
| (*E*)-β-Ocimene* | | | 1.09 | 0.03 | 0.0022 | 0.0013 | sweet, herbal | antibacterial^31^ |
| 5-Methylfurfural | | | 1.07 | 0.01 | 0.0010 | 0.0006 | sweet, caramel |  |
| 4* | | | 1.07 | 0.03 | 0.0023 | 0.0018 |  |  |
|  |  | **Summer** | | | | | | |
| 210 | | | 2.63 | 0.0001 | - | 0.0005 |  |  |
| 224 | | | 2.59 | 0.0001 | - | 0.0021 |  |  |
| 77 | | | 2.42 | 0.0001 | - | 0.0004 |  |  |
| γ-Octanolactone | | | 2.27 | 0.0001 | 0.0002 | 0.0008 | sweet, coconut |  |
| 172 | | | 2.15 | 0.0001 | 0.0003 | 0.0013 |  |  |
| 3,5,5-Trimethylcyclohex-3-en-1-ol | | | 2.15 | 0.0001 | - | 0.0003 |  |  |
| 2-Methyldecane | | | 2.12 | 0.0001 | 0.0001 | 0.0007 |  |  |
| Butyl butanoate | | | 1.84 | 0.0002 | 0.0008 | 0.0048 | sweet, fruit, fatty |  |
| 2-Ethylhexanoic acid* | | | 1.98 | 0.0001 | 0.0001 | 0.0004 |  |  |
| 1-Octen-3-ol | | | 1.92 | 0.0001 | 0.0023 | 0.0066 | mushroom |  |
| Butyl propanoate | | | 1.92 | 0.0001 | 0.0014 | 0.0048 | earthy, fruity |  |
| (2*E*,4*E*)-Heptadienal | | | 1.91 | 0.0004 | 0.0117 | 0.0260 | fatty, oily, fishy |  |
| 166 | | | 1.88 | 0.0004 | 0.0033 | 0.0071 |  |  |
| 171 | | | 1.83 | 0.0005 | 0.0003 | 0.0011 |  |  |
| (2*E*,4*Z*)-Heptadienal | | | 1.83 | 0.0001 | 0.0069 | 0.0250 | fatty, oily, fishy |  |
| (2*E*)-Heptenal | | | 1.82 | 0.0002 | 0.0012 | 0.0021 | green, fatty | antimicrobial ^36^ |
| (2*E*,4*E*)-Decadienal | | | 1.80 | 0.0001 | 0.0003 | 0.0017 | fatty, meaty |  |
| 19 | | | 1.79 | 0.0006 | 0.0005 | 0.0007 |  |  |
| 58 | | | 1.79 | 0.001 | 0.0038 | 0.0070 |  |  |
| 33 | | | 1.77 | 0.0005 | 0.0009 | 0.0018 |  |  |
| Sabina ketone | | | 1.76 | 0.0004 | 0.0005 | 0.0008 |  |  |
| (2*E*)-Octenal | | | 1.69 | 0.0002 | 0.0028 | 0.0053 | green, fatty | antimicrobial^36^ |
| 218* | | | 1.67 | 0.0004 | 0.0007 | 0.0012 |  |  |
| Isoelemicin | | | 1.66 | 0.0002 | 0.0001 | 0.0008 | spice | - |
| Butyl acrylate | | | 1.66 | 0.0001 | 0.0011 | 0.0068 | fruity, spicy | - |
| 1-Octen-3-one | | | 1.66 | 0.0006 | 0.0005 | 0.0008 | mushroom | - |
| Heptanal | | | 1.63 | 0.003 | 0.0031 | 0.0044 | fruity, grassy | antistress^48^ |
| 2-Methylbenzaldehyde | | | 1.62 | 0.006 | 0.0002 | 0.0003 | cherry | antiviral^6^ |
| 147 | | | 1.60 | 0.005 | 0.0008 | 0.0012 |  |  |
| Camphor | | | 1.60 | 0.008 | 0.0007 | 0.0017 | camphor, medicinal | antibacterial^31^ anti-inflammatory^22^ |
| 2,2,6-Trimethylcyclohexanone | | | 1.57 | 0.003 | 0.0034 | 0.0051 | floral, honey |  |
| (2*Z*)-Octen-1-ol* | | | 1.56 | 0.002 | 0.0006 | 0.0011 |  |  |
| 181 | | | 1.55 | 0.005 | 0.0015 | 0.0040 |  |  |
| Octadecane* | | | 1.54 | 0.003 | 0.0026 | 0.0039 |  |  |
| 177 | | | 1.54 | 0.003 | 0.0010 | 0.0039 |  |  |
| 2,6-Dimethyl-3,7-octadiene-2,6-diol* | | | 1.52 | 0.001 | 0.0003 | 0.0012 | fruity, herbal |  |
| 3-Methylacetophenone | | | 1.50 | 0.01 | 0.0005 | 0.0008 |  |  |
| γ-Butyrolactone | | | 1.49 | 0.007 | 0.0020 | 0.0027 | sweet, fatty, oily |  |
| (3*E*,5*E*)-Octadien-2-one | | | 1.47 | 0.0007 | 0.0009 | 0.0025 | grassy, fruity |  |
| 60 | | | 1.46 | 0.001 | 0.0049 | 0.0138 |  |  |
| Hexanoic acid | | | 1.46 | 0.003 | 0.0011 | 0.0021 | sweaty, cheesy |  |
| 87* | | | 1.44 | 0.01 | 0.0001 | 0.0003 |  |  |
| Dihydroactinidiolide | | | 1.44 | 0.008 | 0.0197 | 0.0322 | fruity, woody |  |
| Hexadecane | | | 1.44 | 0.003 | 0.0032 | 0.0050 |  |  |
| Heptanol | | | 1.43 | 0.001 | 0.0006 | 0.0009 | herbal, musty | cardioprotective^49^ |
| Pentanoic acid | | | 1.40 | 0.01 | 0.0005 | 0.0007 | sweaty, rancid |  |
| (4*Z*)-Heptenal* | | | 1.40 | 0.01 | 0.0004 | 0.0007 | oily, fatty, green |  |
| 167* | | | 1.39 | 0.003 | 0.0016 | 0.0024 |  |  |
| 141* | | | 1.38 | 0.005 | 0.0009 | 0.0026 |  |  |
| 36* | | | 1.37 | 0.004 | 0.0009 | 0.0019 |  |  |
| 221 | | | 1.35 | 0.007 | 0.0006 | 0.0013 |  |  |
| (3*Z*)-Hexenyl isovalerate* | | | 1.35 | 0.02 | 0.0001 | 0.0003 | green, fruity |  |
| (2*E*)-Hexenal | | | 1.34 | 0.004 | 0.0001 | 0.0003 | green, fruity, fatty | antimicrobial^36^ |
| 2-Phenoxyethanol* | | | 1.32 | 0.002 | 0.0006 | 0.0015 | mild rose, metallic | antiseptic^38^ |
| 140* | | | 1.30 | 0.01 | 0.0003 | 0.0046 |  |  |
| Nonadecane* | | | 1.30 | 0.02 | 0.0015 | 0.0020 |  |  |
| 90* | | | 1.29 | 0.005 | 0.0006 | 0.0013 |  |  |
| 116 | | | 1.29 | 0.01 | 0.0001 | 0.0002 |  |  |
| 127* | | | 1.28 | 0.02 | 0.0003 | 0.0007 |  |  |
| α-Ionone | | | 1.26 | 0.01 | 0.0046 | 0.0071 | woody, violet, berry |  |
| (2*E*)-Octen-1-ol* | | | 1.24 | 0.04 | 0.0009 | 0.0012 |  |  |
| 4-Vinylanisole | | | 1.22 | 0.0001 | - | 0.0005 | green, herbal, nutty |  |
| 156 | | | 1.22 | 0.005 | 0.0001 | 0.0005 |  |  |
| 2-Ethylhexanol | | | 1.22 | 0.03 | 0.0104 | 0.0140 | green, oily, citrus |  |
| Octanal* | | | 1.18 | 0.02 | 0.0030 | 0.0045 | green, fatty, citrus |  |
| 2-Pentylfuran | | | 1.13 | 0.03 | 0.0008 | 0.0012 | fruity, green, earthy |  |
| 148* | | | 1.12 | 0.02 | 0.0007 | 0.0012 |  |  |
| 138* | | | 1.11 | 0.04 | 0.0014 | 0.0018 |  |  |
| 1-Methylpyrrolidinone* | | | 1.10 | 0.0002 | 0.0002 | 0.0007 |  |  |
| Pentadecane* | | | 1.09 | 0.01 | 0.0012 | 0.0015 |  |  |
| Tetradecane | | | 1.07 | 0.04 | 0.0011 | 0.0013 |  |  |
| (*E*)-β-Ionone | | | 1.06 | 0.03 | 0.0403 | 0.0552 | woody, floral, berry | anticancer^50^ antibacterial^7^ |
| Borneol | | | 1.01 | 0.0001 | 0.0005 | 0.0020 | camphor, woody | antibacterial^31^ antioxidant^13^ analgesic,  anti-inflammatory, anesthetic^51^ |

^a^Aroma information obtained from the Good Scents Company^39^ unless otherwise noted. *Compound is affected by more than one environmental factor. Numbers in the compound column refer to unknown compounds.

**Table S3.** Statistically important metabolites in 2014-2016 Yunnan tea

| **Compound** | **VIP** | **p-value** | **RPA**  **2014** | **RPA**  **2015** | **RPA**  **2016** | **Aroma** | **Health Property** |
| --- | --- | --- | --- | --- | --- | --- | --- |
| **2014** | | | | | | | |
| 22 | 2.44 | 0.0001 | 0.0041 | 0.0027 | 0.0010 |  |  |
| Fokienol | 2.40 | 0.0001 | 0.0040 | 0.0014 | 0.0007 |  |  |
| Pyridine | 2.33 | 0.0001 | 0.0025 | 0.0014 | 0.0007 | fishy, sour |  |
| Indane | 2.30 | 0.0001 | 0.0010 | 0.0003 | 0.0003 |  |  |
| 159 | 2.28 | 0.0001 | 0.0010 | 0.0006 | 0.0004 |  |  |
| Benzyl alcohol | 2.22 | 0.0001 | 0.0019 | 0.0014 | 0.0006 | floral, cherry | antioxidant^20^ |
| Indene | 2.16 | 0.0001 | 0.0006 | 0.0003 | 0.0003 |  |  |
| 2,4-Dimethylheptane | 2.03 | 0.0001 | 0.0036 | 0.0023 | 0.0016 |  |  |
| α-Calacorene | 2.02 | 0.0001 | 0.0012 | 0.0006 | 0.0003 | woody | antibacterial, antioxidant^13^ |
| β-Calacorene* | 1.98 | 0.0002 | 0.0010 | 0.0006 | 0.0004 |  |  |
| Muurola-4,1014-dien-1-β-ol* | 1.93 | 0.0001 | 0.0024 | 0.0012 | 0.0008 |  |  |
| Viridene | 1.90 | 0.001 | 0.0029 | 0.0005 | 0.0006 |  |  |
| (3*Z*)-Hexenyl acetate* | 1.90 | 0.0001 | 0.0032 | 0.0015 | 0.0010 | green, sweet, fruity |  |
| α-Phellandrene | 1.83 | 0.0001 | 0.0011 | 0.0006 | 0.0003 | citrus, terpene, green | analgesic, anti-inflammatory^52^ antibacterial^31^ analgesic^32^ |
| Methyl benzoate* | 1.83 | 0.0002 | 0.0046 | 0.0023 | 0.0021 | cherry, phenolic |  |
| Benzyl acetate | 1.80 | 0.0001 | 0.0035 | 0.0003 | 0.0004 | sweet, floral, fruity | antifungal^18^ |
| 158* | 1.78 | 0.0001 | 0.0012 | 0.0008 | 0.0003 |  |  |
| 44 | 1.76 | 0.0001 | 0.0017 | 0.0012 | 0.0010 |  |  |
| 1,2,4-Trimethylbenzene | 1.75 | 0.0005 | 0.0016 | 0.0013 | 0.0012 | plastic |  |
| Theaspirane A* | 1.74 | 0.0001 | 0.0015 | 0.0011 | 0.0005 | tea, herbal, honey |  |
| Carvone | 1.73 | 0.0001 | 0.0017 | 0.0006 | 0.0006 | spearmint, anise | anticonvulsant, analgesic^51^ antimicrobial, anticancer^53^ |
| 185 | 1.73 | 0.003 | 0.0054 | 0.0021 | 0.0020 |  |  |
| 4-Methyloctane | 1.72 | 0.0009 | 0.0010 | 0.0006 | 0.0005 |  |  |
| 169 | 1.70 | 0.0001 | 0.1638 | 0.0335 | 0.0618 |  |  |
| Theaspirane B* | 1.69 | 0.0002 | 0.0041 | 0.0030 | 0.0013 | tea, herbal, honey |  |
| Heptadecane | 1.66 | 0.0006 | 0.0060 | 0.0035 | 0.0034 |  |  |
| *cis*-Calamenene* | 1.65 | 0.003 | 0.0005 | 0.0003 | 0.0002 | herbal, spicy | antimalarial^28^ antitumor^29^ |
| Homomenthyl salicylate | 1.65 | 0.0007 | 0.0015 | 0.0010 | 0.0010 | mild menthol |  |
| Terpinolene | 1.64 | 0.0004 | 0.0010 | 0.0005 | 0.0005 | woody, terpene | antibacterial^31^ |
| α-Terpinene | 1.63 | 0.001 | 0.0006 | 0.0004 | 0.0003 | citrus, woody | antibacterial^31^ antiviral^34^ |
| Octadecane* | 1.63 | 0.002 | 0.0044 | 0.0029 | 0.0025 |  |  |
| (*Z*)-Herboxide* | 1.59 | 0.0007 | 0.0035 | 0.0017 | 0.0015 | herbal, woody |  |
| Fluorene | 1.59 | 0.0002 | 0.009 | 0.0006 | 0.0007 |  |  |
| 89 | 1.59 | 0.0001 | 0.0018 | 0.0008 | 0.0009 |  |  |
| (*E*)-Herboxide* | 1.59 | 0.001 | 0.0028 | 0.0017 | 0.0015 | herbal, woody |  |
| *epi*-α-Cadinol* | 1.59 | 0.003 | 0.0123 | 0.0082 | 0.0043 | herbal | antibacterial^12^ anticancer^21^  anti-inflammatory^22^ |
| (3*Z*)-Hexenyl butanoate | 1.59 | 0.0001 | 0.0020 | 0.0007 | 0.0004 | fruity, green |  |
| p-tert-Butylphenol | 1.57 | 0.0001 | 0.0025 | 0.0005 | 0.0011 | earthy, leathery |  |
| 153 | 1.56 | 0.009 | 0.0004 | 0.0002 | 0.0002 |  |  |
| m-tert-Butylphenol | 1.54 | 0.0001 | 0.0025 | 0.0005 | 0.0011 |  |  |
| Acetophenone | 1.54 | 0.0003 | 0.0072 | 0.0050 | 0.0053 | floral, almond |  |
| 190 | 1.53 | 0.03 | 0.0021 | 0.0005 | 0.0005 |  |  |
| 21 | 1.52 | 0.0001 | 0.0052 | 0.0030 | 0.0035 |  |  |
| 18 | 1.52 | 0.02 | 0.0003 | 0.0002 | 0.0002 |  |  |
| 4-tert-Butylphenylacetone | 1.52 | 0.0001 | 0.0015 | 0.0004 | 0.0005 |  |  |
| 30 | 1.51 | 0.0008 | 0.0060 | 0.0039 | 0.0041 |  |  |
| *cis*-Methyl dihydrojasmonate | 1.49 | 0.0001 | 0.0100 | 0.0014 | 0.0024 | floral, jasmine |  |
| Cadalene* | 1.48 | 0.009 | 0.0003 | 0.0002 | 0.0002 |  | antibacterial, antioxidant^13^ |
| 88 | 1.48 | 0.0001 | 0.0013 | 0.0004 | 0.0007 |  |  |
| α-Amorphene* | 1.46 | 0.001 | 0.0005 | 0.0003 | 0.0003 |  |  |
| Limonene | 1.45 | 0.001 | 0.0039 | 0.0020 | 0.0021 | lemon, orange | antibacterial^31^cardioprotective^33^  anti-inflammatory, analgesic^32^ |
| β-Homocyclocitral* | 1.44 | 0.0008 | 0.0014 | 0.0008 | 0.0009 | camphor, cooling |  |
| Benzyl nitrile* | 1.43 | 0.009 | 0.0060 | 0.0025 | 0.0014 | floral^41^ |  |
| tert-Pentyl acetate | 1.43 | 0.01 | 0.0010 | 0.0008 | 0.0007 |  |  |
| 6-Methyl-5-hepten-2-one | 1.41 | 0.0002 | 0.0036 | 0.0022 | 0.0022 | fruity, green, musty |  |
| 29 | 1.41 | 0.001 | 0.0038 | 0.0029 | 0.0030 |  |  |
| 128* | 1.40 | 0.01 | 0.0005 | 0.0004 | 0.0003 |  |  |
| 202* | 1.38 | 0.002 | 0.0006 | 0.0003 | 0.0003 |  |  |
| Hotrienol | 1.38 | 0.009 | 0.0750 | 0.0537 | 0.0254 | floral, woody, spice |  |
| γ-Terpinene | 1.36 | 0.01 | 0.0007 | 0.0005 | 0.0004 | citrus, terpene | antibacterial^31^ antiviral^34^ |
| 2,2,4-Trimethylhexane* | 1.35 | 0.03 | 0.0016 | 0.0012 | 0.0010 |  |  |
| Isoborneol* | 1.35 | 0.002 | 0.0008 | 0.0005 | 0.0005 | camphor, herbal | antiviral, antibacterial^24^ |
| (*E*)-β-Ocimene* | 1.34 | 0.02 | 0.0026 | 0.0015 | 0.0011 | sweet, herbal | antibacterial^31^ |
| 1,2,3-Trimethylbenzene | 1.33 | 0.0002 | 0.0007 | 0.0004 | 0.0005 |  |  |
| 178 | 1.33 | 0.0008 | 0.0295 | 0.0098 | 0.0149 |  |  |
| *epi*-α-Murrolol* | 1.32 | 0.01 | 0.0056 | 0.0045 | 0.0027 | herbal, spicy | antibacterial^12^ antioxidant^13^ |
| 2-Ethylhexyl salicylate | 1.31 | 0.007 | 0.0026 | 0.0019 | 0.0019 | floral, sweet |  |
| *p*-Cymene | 1.29 | 0.007 | 0.0013 | 0.0009 | 0.0010 | citrus, terpene, woody | antibacterial^31^ hypotensive^33^ antiviral^34^ analgesic^32^ |
| 2-Cyclopenten-1-one | 1.28 | 0.03 | 0.0015 | .0012 | 0.0011 |  | anti-inflammatory^54^ |
| Safranal* | 1.28 | 0.04 | 0.0045 | 0.0033 | 0.0024 | sweet, herbal | antinociceptive^42^ antimicrobial^43^ |
| Geranial | 1.25 | 0.0003 | 0.0051 | 0.0016 | 0.0020 | citrus, mint | antibacterial^31^ antifungal^55^ |
| Methyl salicylate | 1.23 | 0.01 | 0.0337 | 0.0170 | 0.0091 | wintergreen | anti-inflammatory, analgesic^56^ |
| 103 | 1.22 | 0.0005 | 0.0027 | 0.0012 | 0.0016 |  |  |
| 2,2,5,5-Tetramethyltetrahydrofuran | 1.21 | 0.02 | 0.0017 | 0.0013 | 0.0011 |  |  |
| 111 | 1.21 | 0.004 | 0.0034 | 0.0028 | 0.0012 |  |  |
| 2-Methylnaphthalene | 1.17 | 0.0001 | 0.0029 | 0.0016 | 0.0022 | herbal |  |
| 201 | 1.17 | 0.0004 | 0.0023 | 0.0014 | 0.0018 |  |  |
| β-Cyclocitral* | 1.16 | 0.005 | 0.0095 | 0.0057 | 0.0065 | sweet, herbal |  |
| Geranylacetone | 1.15 | 0.0001 | 0.0028 | 0.0012 | 0.0019 | floral, green, earthy |  |
| 3-Phenyl-2-butanone | 1.14 | 0.007 | 0.0006 | 0.0004 | 0.0004 |  |  |
| 3,4-Diethyl-1,1'-biphenyl | 1.13 | 0.0001 | 0.0009 | 0.0004 | 0.0006 |  |  |
| Nonadecane* | 1.12 | 0.01 | 0.0023 | 0.0014 | 0.0016 |  |  |
| Cumene | 1.10 | 0.005 | 0.0004 | 0.0003 | 0.0003 |  |  |
| 2,6-Dimethylcyclohexanol | 1.10 | 0.0003 | 0.0026 | 0.0010 | 0.0018 |  | anesthetic^57^ |
| Linalool 3,7-oxide | 1.02 | 0.004 | 0.0017 | 0.0013 | 0.0013 | floral, woody |  |
| Methyl pyruvate* | 1.02 | 0.002 | 0.0003 | 0.0002 | 0.0002 |  |  |
| 1-Methylnaphthalene | 1.00 | 0.0001 | 0.0016 | 0.0009 | 0.0012 | camphor, medicinal |  |
| **2016** | | | | | | | |
| 4-Ethyl-2-methoxyphenol | 2.30 | 0.0001 | - | 0.0002 | 0.0004 | smoky, phenolic |  |
| 82 | 2.04 | 0.0001 | - | - | 0.0131 |  |  |
| Pyranone | 2.02 | 0.0001 | 0.0003 | 0.0006 | 0.0014 |  |  |
| Tetradecanoic acid | 1.89 | 0.0002 | 0.0024 | 0.0049 | 0.0062 | coconut, waxy | antimicrobial^58^ |
| 174 | 1.87 | 0.0001 | 0.0014 | 0.0006 | 0.0130 |  |  |
| 2,4-Dimethylbenzaldehyde* | 1.84 | 0.0008 | 0.0005 | 0.0005 | 0.0009 | almond, cherry | antiviral^6^ |
| Catechol | 1.82 | 0.0006 | 0.0006 | 0.0018 | 0.0034 |  | antioxidant,  anti-inflammatory^59^ |
| 175 | 1.80 | 0.0001 | 0.0007 | 0.0003 | 0.0041 |  |  |
| 85 | 1.77 | 0.0001 | 0.0009 | 0.0008 | 0.0016 |  |  |
| 2-Hydroxy-γ-butyrolactone | 1.73 | 0.002 | 0.0010 | 0.0014 | 0.0126 |  |  |
| Phorone | 1.68 | 0.0002 | 0.0001 | - | 0.0037 |  |  |
| γ-Heptalactone | 1.60 | 0.01 | 0.0004 | 0.0005 | 0.0012 | sweet, nutty |  |
| 4-Methyl-3-penten-2-one* | 1.59 | 0.004 | 0.0062 | 0.0039 | 0.0221 | sweet, earthy |  |
| 2-Hydroxy-2-cyclopenten-1-one | 1.56 | 0.0002 | 0.0019 | 0.0025 | 0.0045 | maple, caramel |  |
| 26* | 1.55 | 0.0008 | 0.0017 | 0.0025 | 0.0131 |  |  |
| Furfural | 1.55 | 0.01 | 0.0045 | 0.0050 | 0.0076 | sweet, bready |  |
| 83 | 1.54 | 0.005 | - | 0.0001 | 0.0002 |  |  |
| 70 | 1.54 | 0.0003 | 0.0079 | 0.0109 | 0.0185 |  |  |
| 2(5H)-Furanone | 1.51 | 0.001 | 0.0036 | 0.0039 | 0.0072 | buttery |  |
| 1,2-Cyclopentanedione | 1.49 | 0.0003 | 0.0020 | 0.0024 | 0.0046 |  |  |
| 93 | 1.49 | 0.01 | 0.0009 | 0.0026 | 0.0055 |  |  |
| *p*-Acetyltoluene | 1.46 | 0.003 | 0.0011 | 0.0011 | 0.0020 | sweet, creamy |  |
| 140* | 1.43 | 0.001 | 0.0003 | 0.0003 | 0.0067 |  |  |
| Dodecane | 1.42 | 0.01 | 0.0007 | 0.0007 | 0.0010 |  |  |
| γ-Nonalactone | 1.39 | 0.0002 | 0.0026 | 0.0021 | 0.0035 | sweet, coconut |  |
| 218* | 1.39 | 0.03 | 0.0007 | 0.0009 | 0.0012 |  |  |
| Octadecanoic acid | 1.36 | 0.01 | 0.0120 | 0.0179 | 0.0344 |  | antimicrobial^58^ |
| Dodecanoic acid | 1.09 | 0.03 | 0.0042 | 0.0042 | 0.0063 | coconut, fatty | cardioprotective^60^antibacterial,  anti-inflammatory^61^ |
| Benzeneacetaldehyde | 1.06 | 0.0003 | 0.0035 | 0.0023 | 0.0051 | floral, honey |  |
| Benzothiazole | 1.07 | 0.009 | 0.0021 | 0.0017 | 0.0030 | sulfur, rubbery |  |
| (2*E*)-Octen-1-ol* | 1.02 | 0.02 | 0.0010 | 0.0009 | 0.0014 | green, fatty |  |
| **2015/2016** | | | | | | | |
| Pentadecanoic acid | 1.66 | 0.001 | 0.0005 | 0.0010 | 0.0010 | waxy |  |
| 20 | 1.58 | 0.001 | 0.0028 | 0.0044 | 0.0049 |  |  |
| 80* | 1.57 | 0.01 | 0.0006 | 0.0011 | 0.0012 |  |  |
| 141* | 1.48 | 0.003 | 0.0001 | 0.0026 | 0.0025 |  |  |
| Tridecane | 1.39 | 0.01 | 0.0006 | 0.0008 | 0.0009 |  |  |
| 94 | 1.37 | 0.0001 | 0.0008 | 0.0023 | 0.0019 |  |  |
| Tetradecanamide | 1.35 | 0.0001 | 0.0037 | 0.0113 | 0.0103 |  |  |
| Tridecanoic acid* | 1.35 | 0.009 | 0.0010 | 0.0015 | 0.0016 | waxy, woody |  |
| 187 | 1.30 | 0.0001 | 0.0004 | 0.0027 | 0.0025 |  |  |
| 23 | 1.24 | 0.006 | 0.0447 | 0.0680 | 0.0671 |  |  |
| 75 | 1.17 | 0.01 | - | 0.0003 | 0.0003 |  |  |
| 220 | 1.14 | 0.0001 | 0.0006 | 0.0042 | 0.0028 |  |  |
| Dodecanamide* | 1.12 | 0.0006 | 0.0058 | 0.0109 | 0.0098 |  |  |
| 172* | 1.06 | 0.01 | 0.0005 | 0.0008 | 0.0010 |  |  |
| Hexadecanoic acid | 1.04 | 0.04 | 0.0351 | 0.0428 | 0.0595 | slight waxy | antimicrobial^58^ |
| **2014/2015** | | | | | | | |
| Isopropyl myristate | 1.84 | 0.0001 | 0.0004 | 0.0003 | 0.0002 |  |  |
| 48 | 1.82 | 0.0001 | 0.0019 | 0.0014 | 0.0009 |  |  |
| 2-Methylbutanoic acid | 1.73 | 0.0006 | 0.0004 | 0.0004 | 0.0001 | cheesy, fruity |  |
| 35* | 1.59 | 0.004 | 0.0188 | 0.0154 | 0.0118 |  |  |
| (*Z*)-Methyl *epi*-jasmonate | 1.56 | 0.003 | 0.0031 | 0.0018 | 0.0010 | sweet, floral |  |
| (*Z*)-β-Ocimene | 1.55 | 0.0001 | 0.0012 | 0.0006 | 0.0003 | herbal | antibacterial^31^ |
| 148* | 1.37 | 0.001 | 0.0013 | 0.0012 | 0.0004 |  |  |
| δ-Decalactone | 1.25 | 0.01 | 0.0039 | 0.0034 | 0.0018 | coconut, peach |  |
| Butanoic acid | 1.23 | 0.001 | 0.0007 | 0.0008 | 0.0005 | cheesy, sweaty |  |
| Myrtenol | 1.23 | 0.007 | 0.0034 | 0.0029 | 0.0021 | pine, sweet, minty | antibacterial^31^ hypotensive^33^ analgesic, anti-inflammatory^52^ |
| 113 | 1.22 | 0.005 | 0.0009 | 0.0007 | 0.0004 |  |  |
| Jasmine lactone* | 1.20 | 0.003 | 0.0069 | 0.0064 | 0.0017 | jasmine, fruity |  |
| 4-2,6,6-Trimethylcyclohexa-1,5-dienylbut-3-en-2-one | 1.20 | 0.003 | 0.0014 | 0.0013 | 0.0010 |  |  |
| 36* | 1.19 | 0.04 | 0.0018 | 0.0016 | 0.0007 |  |  |
| Bornylene | 1.14 | 0.02 | 0.0072 | 0.0065 | 0.0029 |  |  |
| Isovaleric acid | 1.11 | 0.001 | 0.0012 | 0.0013 | 0.0005 | cheesy, fruity |  |
| α-Cadinol | 1.09 | 0.04 | 0.0095 | 0.0085 | 0.0040 | herbal, woody | antibacterial, antioxidant^12^  anti-inflammatory^22^ |
| Salicylaldehyde | 1.04 | 0.01 | 0.0003 | 0.0003 | 0.0002 | wintergreen |  |
| *p*-Cymenene | 1.04 | 0.03 | 0.0003 | 0.0003 | 0.0002 | spicy, medicinal |  |
| (4*Z*)-Heptenal* | 1.04 | 0.04 | 0.0006 | 0.0006 | 0.0004 | oily, fatty, green |  |
| **2015** | | | | | | | |
| 134 | 1.14 | 0.0002 | - | 0.0014 | 0.0007 |  |  |
| 170 | 1.02 | 0.0002 | - | 0.0019 | 0.0009 |  |  |

^a^Aroma information obtained from the Good Scents Company^39^ unless otherwise noted. *Compound is affected by more than one environmental factor. Numbers in the compound column refer to unknown compounds.

**Table S4.** Statistically important metabolites in spring and summer Fujian tea

| **Compound** | **VIP** | **p-value** | **RPA**  **Spring** | **RPA**  **Summer** | **Aroma** | **Health Property** | |
| --- | --- | --- | --- | --- | --- | --- | --- |
| **Spring** | | | | | | |  |
| N-Ethylsuccinimide | 2.78 | 0.0001 | 0.0006 | - |  |  | |
| 2-Ethylfuran | 2.35 | 0.0001 | 0.0005 | - | sweet, earthy, musty |  | |
| 4-keto-Isophorone | 1.76 | 0.0001 | 0.0004 | - | floral, woody |  | |
| Isomenthone | 1.71 | 0.0001 | 0.0006 | - | sweet, peppermint |  | |
| Isoborneol | 2.85 | 0.0001 | 0.0012 | 0.0005 | camphor, herbal | antiviral, antibacterial^24^ | |
| 18 | 2.70 | 0.0001 | 0.0003 | 0.0001 |  |  | |
| 153 | 2.65 | 0.0001 | 0.0007 | 0.0001 |  |  | |
| 6-Methyl-2-heptanone | 2.59 | 0.0001 | 0.0013 | 0.0009 | camphoraceous |  | |
| 3-Heptanone | 2.44 | 0.0001 | 0.0022 | 0.0013 | green, fatty, fruity |  | |
| Coumarin | 2.36 | 0.0001 | 0.0255 | 0.0079 | sweet, hay | antidiabetic^62^ anticancer  anti-inflammatory, antipyretic, ^63^ | |
| Pyrethrone | 2.21 | 0.0001 | 0.0016 | 0.0009 |  |  | |
| 89 | 2.21 | 0.0001 | 0.0031 | 0.0015 |  |  | |
| 168* | 2.19 | 0.0001 | 0.0006 | 0.0003 |  |  | |
| 202 | 2.16 | 0.0001 | 0.0010 | 0.0004 |  |  | |
| 168 | 2.16 | 0.0004 | 0.0006 | 0.0003 |  |  | |
| Butyl acetate* | 2.10 | 0.0004 | 0.0024 | 0.0008 | sweet, fruity |  | |
| 54 | 1.91 | 0.002 | 0.0003 | 0.0001 |  |  | |
| Geranic acid | 1.90 | 0.002 | 0.0044 | 0.0018 | green, woody |  | |
| Styrene | 1.89 | 0.0009 | 0.0480 | 0.0320 | sweet, floral, balsamic |  | |
| 2,6-Dimethylcyclohexanol | 1.88 | 0.0008 | 0.0042 | 0.0023 | roasted, phenolic | anesthetic^57^ | |
| (*E*)-β-Ocimene | 1.85 | 0.0005 | 0.0009 | 0.0004 | sweet, herbal | antibacterial^31^ | |
| (3*E*)-Methylbutanal oxime | 1.83 | 0.002 | 0.0006 | 0.0003 |  |  | |
| 2,2,6-Trimethylcyclohexanone | 1.82 | 0.005 | 0.0076 | 0.0051 | floral, honey |  | |
| Menthone | 1.78 | 0.0005 | 0.0017 | 0.0004 | green, minty | antibacterial^31^  anti-inflammatory^40^ | |
| 7-Methoxycoumarin | 1.75 | 0.001 | 0.0095 | 0.0052 | sweet, balsamic | anticancer^1^ antinociceptive^2^  anti-inflammatory^3^ | |
| 160 | 1.67 | 0.0008 | 0.0002 | - |  |  | |
| Ethylbenzene | 1.66 | 0.004 | 0.0057 | 0.0043 |  |  | |
| 102 | 1.65 | 0.0008 | 0.0007 | 0.0003 |  |  | |
| 115 | 1.63 | 0.008 | 0.0011 | 0.0006 |  |  | |
| Methyl o-anisate | 1.63 | 0.005 | 0.0016 | 0.0009 | floral, fruity |  | |
| Fluoranthene | 1.62 | 0.005 | 0.0014 | 0.0010 |  |  | |
| 1-Ethyl-1H-pyrrole | 1.62 | 0.0008 | 0.0006 | - | roasted |  | |
| 201 | 1.58 | 0.01 | 0.0024 | 0.0017 |  |  | |
| (*Z*)-Jasmone | 1.54 | 0.0008 | 0.0378 | 0.0135 | floral, jasmine | antibacterial^7^ anticancer^8^ | |
| 76 | 1.53 | 0.006 | 0.0038 | 0.0012 |  |  | |
| 2-Heptanone | 1.51 | 0.007 | 0.0018 | 0.0013 | fruity, herbal, sweet |  | |
| α-Amorphene | 1.49 | 0.03 | 0.0007 | 0.0005 |  |  | |
| 214 | 1.49 | 0.009 | 0.0008 | - |  |  | |
| α-Cyclocitral | 1.48 | 0.006 | 0.0008 | 0.0006 |  |  | |
| allo-Ocimene | 1.48 | 0.009 | 0.0001 | - | sweet, floral, peppery |  | |
| Perilla aldehyde | 1.48 | 0.01 | 0.0009 | 0.0006 | fruity, grassy | anti-inflammatory, antioxidant, antidepressant^64^ | |
| 1-Ethyl-1H-pyrrole-2-carboxaldehyde | 1.47 | 0.01 | 0.0053 | 0.0017 | roasted, smoky |  | |
| γ-Terpinene | 1.43 | 0.02 | 0.0002 | 0.0001 | citrus, terpene, sweet | antibacterial^31^ antiviral^34^ | |
| 65 | 1.28 | 0.005 | 0.0006 | 0.0004 |  |  | |
| 211* | 1.27 | 0.003 | 0.0007 | 0.0001 |  |  | |
| *cis*-Methyl dihydrojasmonate | 1.27 | 0.03 | 0.0028 | 0.0021 | floral, jasmine, green |  | |
| Tetradecane | 1.24 | 0.02 | 0.0014 | 0.0011 |  |  | |
| β-Homocyclocitral | 1.10 | 0.03 | 0.0013 | 0.0011 | camphor, cooling, woody |  | |
| **Summer** | | | | | | |  |
| Cubebol | 2.34 | 0.0001 | - | 0.0006 | spicy, minty |  | |
| Spathulenol | 2.25 | 0.0001 | - | 0.0009 | earthy, herbal | antiproliferative, antioxidant,  anti-inflammatory, antimicrobial^65^ | |
| beta-Cubebene | 2.17 | 0.0001 | - | 0.0006 | fruity, citrus |  | |
| 194 | 1.47 | 0.009 | - | 0.0001 |  |  | |
| 2-Hydroxy-2-cyclopenten-1-one | 2.54 | 0.0001 | 0.0002 | 0.0018 | maple, caramel |  | |
| Nerol oxide | 2.39 | 0.0001 | 0.0001 | 0.0005 | green, herbal |  | |
| Methyl anthranilate | 2.39 | 0.0001 | 0.0003 | 0.0011 | fruity, grape | antifungal^18^ | |
| Muurola-4,1014-dien-1-β-ol | 2.18 | 0.004 | 0.0010 | 0.0042 |  |  | |
| Cadalene | 2.08 | 0.0008 | 0.0001 | 0.0005 |  | antibacterial, antioxidant^13^ | |
| *cis*-Calamenene | 2.03 | 0.0009 | 0.0004 | 0.0010 | herbal, spicy | antimalarial^28^ antitumor^29^ | |
| 217 | 2.03 | 0.0005 | 0.0002 | 0.0013 |  |  | |
| Bornylene | 1.98 | 0.04 | 0.0008 | 0.0049 |  |  | |
| α-Muurolol | 1.97 | 0.0001 | 0.0005 | 0.0064 |  | antibacterial, antioxidant^12^ | |
| β-Calacorene | 1.95 | 0.001 | 0.0011 | 0.0027 |  |  | |
| alpha-Copaene | 1.90 | 0.0004 | 0.0001 | 0.0005 | woody, spice |  | |
| Caryophyllene oxide | 1.90 | 0.002 | 0.0007 | 0.0014 | woody, spice | anticancer, analgesic,  anti-inflammatory^66^ | |
| Pentanal | 1.89 | 0.001 | 0.0021 | 0.0029 | fruity, fermented |  | |
| α-Calacorene | 1.89 | 0.005 | 0.0014 | 0.0039 | woody | antibacterial, antioxidant^13^ | |
| *epi*-α-Cadinol | 1.89 | 0.01 | 0.0032 | 0.0244 | herbal | antibacterial^12^ anticancer^21^  anti-inflammatory^22^ | |
| 36* | 1.88 | 0.0009 | 0.0011 | 0.0027 |  |  | |
| *epi*-α-Murrolol | 1.87 | 0.02 | 0.0019 | 0.0125 | herbal, spicy | antibacterial^12^ antioxidant^13^ | |
| 195 | 1.85 | 0.003 | - | 0.0008 |  |  | |
| 176 | 1.84 | 0.003 | 0.0006 | 0.0011 |  |  | |
| Quinoline | 1.84 | 0.001 | 0.0006 | 0.0009 | musty, earthy | antimalarial, anticancer, analgesic antibacterial, anticonvulsant, antifungal, anti-inflammatory^67^ | |
| *epi*-Cubebol | 1.83 | 0.0001 | 0.0001 | 0.0007 |  |  | |
| 224 | 1.83 | 0.002 | 0.0004 | 0.0022 |  |  | |
| 112 | 1.82 | 0.003 | 0.0007 | 0.0030 |  |  | |
| 192 | 1.82 | 0.0008 | 0.0007 | 0.0029 |  |  | |
| Benzyl nitrile | 1.80 | 0.001 | 0.0013 | 0.0031 | floral^41^ |  | |
| 96 | 1.79 | 0.0008 | 0.0002 | 0.0017 |  |  | |
| Aniline | 1.77 | 0.0003 | 0.0002 | 0.0007 |  |  | |
| Hotrienol | 1.76 | 0.04 | 0.0089 | 0.0342 | floral, woody, spice |  | |
| 167 | 1.73 | 0.0008 | 0.0024 | 0.0059 |  |  | |
| 111 | 1.65 | 0.01 | 0.0002 | 0.0019 |  |  | |
| 4-Ethylbenzaldehyde | 1.53 | 0.02 | 0.0002 | 0.0005 | bitter, almond |  | |
| 17 | 1.48 | 0.01 | 0.0006 | 0.0008 |  |  | |
| 31* | 1.48 | 0.01 | 0.0020 | 0.0036 |  |  | |
| 154 | 1.48 | 0.02 | 0.0004 | 0.0013 |  |  | |
| Benzophenone | 1.45 | 0.01 | 0.0124 | 0.0257 | fruity, floral, metallic |  | |
| 2-Phenoxyethanol | 1.45 | 0.009 | - | 0.0004 | mild rose, metallic | antiseptic^38^ | |
| 2-Ethylhexyl salicylate | 1.44 | 0.01 | 0.0012 | 0.0016 | floral, sweet |  | |
| (*E*)-β-Damascenone | 1.44 | 0.02 | 0.0003 | 0.0005 | floral, sweet, fruity |  | |
| 79 | 1.39 | 0.04 | 0.0002 | 0.0004 |  |  | |
| 181 | 1.39 | 0.03 | 0.0011 | 0.0027 |  |  | |
| Benzeneacetaldehyde | 1.38 | 0.02 | 0.0016 | 0.0021 | floral, honey |  | |
| 99 | 1.33 | 0.004 | 0.0004 | 0.0018 |  |  | |
| 219 | 1.33 | 0.04 | 0.0004 | 0.0012 |  |  | |
| Dimethyl sulfoxide* | 1.29 | 0.0004 | 0.0010 | 0.0018 | garlic, bitter | antioxidant, analgesic, neuroprotective, cardioprotective,  anti-inflammatory^68^ | |
| 1,2-Cyclopentanedione* | 1.28 | 0.02 | 0.0009 | 0.0018 |  |  | |
| 6 | 1.27 | 0.04 | 0.0010 | 0.0017 |  |  | |
| 123 | 1.26 | 0.03 | 0.0005 | 0.0021 |  |  | |
| 23* | 1.10 | 0.03 | 0.0525 | 0.0776 |  |  | |
| 230 | 1.05 | 0.03 | 0.0004 | 0.0006 |  |  | |

^a^Aroma information obtained from the Good Scents Company^39^ unless otherwise noted. *Compound is affected by more than one environmental factor. Numbers in the compound column refer to unknown compounds.

**Table S5.** Statistically important metabolites in 2014-2016 Fujian tea

| **Compound** | | **VIP** | **p-value** | **RPA**  **2014** | | **RPA**  **2015** | | **RPA**  **2016** | **Aroma** | | | **Health Property** | |
| --- | --- | --- | --- | --- | --- | --- | --- | --- | --- | --- | --- | --- | --- |
| **2016** |  |  | | |  | |  | | |  |  | |  |
| 71 | | 2.70 | 0.0001 | 0.0003 | | 0.0009 | | 0.0023 |  | | |  | |
| 2-Ethylhexanoic acid | | 2.42 | 0.0001 | 0.0001 | | 0.0002 | | 0.0006 |  | | |  | |
| 140 | | 2.33 | 0.0001 | 0.0003 | | 0.0010 | | 0.0059 |  | | |  | |
| 10 | | 2.31 | 0.0001 | 0.0490 | | 0.0725 | | 0.0898 |  | | |  | |
| 86 | | 2.30 | 0.0001 | 0.0007 | | 0.0017 | | 0.0055 |  | | |  | |
| Ethyl 2-methyl butyrate | | 2.28 | 0.0001 | 0.0019 | | 0.0030 | | 0.0037 | sweet, fruity | | |  | |
| 3-Methylacetophenone | | 2.23 | 0.0001 | 0.0005 | | 0.0005 | | 0.0010 |  | | |  | |
| 2-Hydroxy-γ-butyrolactone | | 2.23 | 0.0001 | - | | - | | 0.0012 |  | | |  | |
| 4,4-Dimethyl-2-pentanone | | 2.18 | 0.0001 | 0.0017 | | 0.0024 | | 0.0030 |  | | |  | |
| (*E*)-Isobutyraldehyde oxime | | 2.07 | 0.0006 | 0.0007 | | 0.0010 | | 0.0016 |  | | |  | |
| Camphor | | 2.05 | 0.0001 | 0.0014 | | 0.0013 | | 0.0075 | camphor, medicinal | | | antibacterial^31^ anti-inflammatory^22^ | |
| 1,2-Cyclopentanedione* | | 2.04 | 0.0001 | 0.0004 | | 0.0003 | | 0.0005 |  | | |  | |
| 84 | | 2.01 | 0.0003 | 0.0005 | | 0.0007 | | 0.0020 |  | | |  | |
| 1-Methylpyrrolidinone | | 2.01 | 0.0001 | 0.0002 | | 0.0003 | | 0.0012 |  | | |  | |
| 117 | | 2.00 | 0.0001 | 0.0006 | | 0.0006 | | 0.0013 |  | | |  | |
| 3-Methylpyridine | | 1.99 | 0.0003 | - | | 0.0008 | | 0.0020 | green, earthy, nutty | | |  | |
| 6 | | 1.96 | 0.002 | 0.0007 | | 0.0012 | | 0.0022 |  | | |  | |
| 20 | | 1.95 | 0.0001 | 0.0024 | | 0.0036 | | 0.0062 |  | | |  | |
| Neral | | 1.93 | 0.0001 | 0.0011 | | 0.0013 | | 0.0040 | sweet, lemon | | | antibacterial^31^ antifungal^55^ | |
| Heptanol | | 1.86 | 0.0002 | 0.0009 | | 0.0010 | | 0.0016 | green, herbal, musty | | | cardioprotective^49^ | |
| 23* | | 1.81 | 0.004 | 0.0414 | | 0.0551 | | 0.0987 |  | | |  | |
| Linalool acetate | | 1.78 | 0.0002 | 0.0006 | | 0.0013 | | 0.0048 | sweet, green, floral | | | analgesic^32^ antibacterial^31^ | |
| Cyclohexanone | | 1.75 | 0.0002 | 0.0005 | | 0.0004 | | 0.0008 | minty | | |  | |
| Decane | | 1.75 | 0.002 | 0.0028 | | 0.0038 | | 0.0079 |  | | |  | |
| 114 | | 1.70 | 0.0001 | 0.0007 | | 0.0006 | | 0.0012 |  | | |  | |
| 25H-Furanone | | 1.68 | 0.004 | 0.0020 | | 0.0024 | | 0.0033 | buttery | | |  | |
| Eucalyptol | | 1.66 | 0.009 | 0.0003 | | 0.0005 | | 0.0020 | eucalyptus, sweet | | | antibacterial^31^ analgesic^32^ cardioprotective^33^ antiviral^34^ | |
| Butyl acrylate | | 1.62 | 0.0001 | 0.0035 | | 0.0025 | | 0.0071 | fruity, spicy | | |  | |
| Geranylacetone | | 1.62 | 0.0006 | 0.0021 | | 0.0022 | | 0.0045 | floral, green, earthy | | |  | |
| 5-Methylfurfural | | 1.61 | 0.0001 | 0.0004 | | 0.0003 | | 0.0007 | sweet, caramel | | |  | |
| Octanol | | 1.61 | 0.0004 | 0.0024 | | 0.0024 | | 0.0034 | fruity, green, earthy | | | anesthetic^69^ | |
| (3*Z*)-Hexenyl isovalerate | | 1.61 | 0.0002 | 0.0003 | | 0.0003 | | 0.0007 | green, fruity | | |  | |
| 4-Methyl-3-penten-2-one | | 1.61 | 0.003 | 0.0038 | | 0.0032 | | 0.0080 | sweet, earthy | | |  | |
| α-Pinene | | 1.56 | 0.0004 | 0.0003 | | 0.0003 | | 0.0006 | pine, camphor | | | antibacterial^31^ hypotensive^33^  antiviral^34^ analgesic^32^ | |
| Tridecane | | 1.56 | 0.01 | 0.0006 | | 0.0007 | | 0.0010 |  | | |  | |
| Hexyl acetate | | 1.55 | 0.009 | 0.0004 | | 0.0004 | | 0.0006 | sweet, fruity | | |  | |
| o-Guaiacol | | 1.55 | 0.0001 | 0.0003 | | 0.0001 | | 0.0006 | phenolic, smoky | | |  | |
| 148 | | 1.53 | 0.003 | 0.0006 | | 0.0006 | | 0.0015 |  | | |  | |
| Propanoic acid | | 1.52 | 0.01 | 0.0008 | | 0.0009 | | 0.0011 | cheesy, pungent | | |  | |
| 2-Cyclopentene-1,4-dione | | 1.48 | 0.01 | 0.0003 | | 0.0004 | | 0.0005 |  | | |  | |
| Dimethyl sulfoxide* | | 1.48 | 0.007 | 0.0010 | | 0.0009 | | 0.0023 | garlic, bitter | | | antioxidant, neuroprotective, cardioprotective, analgesic,  anti-inflammatory^68^ | |
| Vanillin | | 1.47 | 0.0005 | 0.0012 | | 0.0009 | | 0.0022 | vanilla | | | antimicrobial, antioxidant, analgesic, antimutagenic, antidepressant^70^ | |
| *p*-Cymene | | 1.47 | 0.02 | 0.0010 | | 0.0012 | | 0.0027 | citrus, terpene, woody | | | antibacterial^31^ hypotensive^33^  antiviral^34^ antioxidant^20^ | |
| Nerol | | 1.47 | 0.02 | 0.0001 | | 0.0001 | | 0.0004 | sweet, floral | | | antifungal^55^ antinociceptive,  anti-inflammatory^71^ | |
| 70 | | 1.46 | 0.003 | 0.0040 | | 0.0061 | | 0.0088 |  | | |  | |
| 2-Nonanone | | 1.42 | 0.02 | 0.0013 | | 0.0014 | | 0.0018 | green, earthy, soapy | | | antimicrobial^72^ | |
| 39 | | 1.37 | 0.03 | 0.0021 | | 0.0029 | | 0.0035 |  | | |  | |
| 6-Methyl-2-heptanol | | 1.35 | 0.005 | 0.0008 | | 0.0008 | | 0.0011 | waxy, fatty, citrus | | |  | |
| 226 | | 1.33 | 0.03 | 0.0010 | | 0.0017 | | 0.0021 |  | | |  | |
| 1-Hydroxy-2-propanone | | 1.33 | 0.02 | 0.0142 | | 0.0187 | | 0.0259 | sweet, caramel | | |  | |
| 203 | | 1.33 | 0.0004 | 0.0056 | | 0.0057 | | 0.0088 |  | | |  | |
| Benzothiazole | | 1.31 | 0.001 | 0.0026 | | 0.0032 | | 0.0050 | sulfury, rubbery | | |  | |
| Limonene | | 1.30 | 0.006 | 0.0014 | | 0.0011 | | 0.0039 | lemon, orange | | | antibacterial^31^ cardioprotective^33^  anti-inflammatory, analgesic^32^ | |
| (2*Z*)-Octen-1-ol | | 1.29 | 0.0003 | 0.0015 | | 0.0010 | | 0.0027 |  | | |  | |
| Norfuraneol | | 1.29 | 0.03 | 0.0020 | | 0.0023 | | 0.0029 | sweet, caramel | | |  | |
| Butyl acetate* | | 1.23 | 0.005 | 0.0006 | | 0.0005 | | 0.0011 | sweet, fruity | | |  | |
| 2-Methoxy-4-vinylphenol | | 1.20 | 0.04 | 0.0005 | | 0.0007 | | 0.0014 | smoky, clove | | | anti-inflammatory^27^ | |
| Isophorone | | 1.18 | 0.02 | 0.0004 | | 0.0003 | | 0.0007 | sweet, woody, cooling | | |  | |
| 2-Ethylhexanol | | 1.14 | 0.006 | 0.0217 | | 0.0182 | | 0.0391 | green, oily, citrus | | |  | |
| 5-Ethyl-2(5H)-furanone | | 1.08 | 0.01 | 0.0061 | | 0.0053 | | 0.0103 | spice | | |  | |
| 75 | | 1.04 | 0.02 | 0.0029 | | 0.0027 | | 0.0055 |  | | |  | |
| 174 | | 1.66 | 0.0001 | 0.0019 | | 0.0002 | | 0.0124 |  | | |  | |
| 175 | | 1.61 | 0.0001 | 0.0010 | | 0.0001 | | 0.0045 |  | | |  | |
| Nonanol | | 1.48 | 0.0001 | 0.0016 | | 0.0011 | | 0.0024 | fatty, orange, floral | | | anesthetic^69^ | |
| Octanal | | 1.38 | 0.0001 | 0.0055 | | 0.0031 | | 0.0088 | green, fatty, citrus | | |  | |
| Nonanal | | 1.35 | 0.0001 | 0.0219 | | 0.0120 | | 0.0342 | cucumber, waxy | | | antifungal^36^ | |
| Butyl butanoate | | 1.18 | 0.0001 | 0.0018 | | 0.0005 | | 0.0031 | sweet, fruit | | |  | |
| **2015/2016** |  |  | | |  | |  | | |  |  | |  |
| 2,2,4-Trimethylhexane | | 2.00 | 0.0002 | 0.0017 | | 0.0025 | | 0.0033 |  | | |  | |
| Terpinen-4-ol | | 1.96 | 0.0001 | 0.0010 | | 0.0022 | | 0.0026 | woody, terpene, cooling | | | hypotensive^33^ antibacterial^31^  anticancer^73^ antiviral^34^ | |
| 91 | | 1.95 | 0.0001 | 0.0053 | | 0.0090 | | 0.0139 |  | | |  | |
| 47 | | 1.94 | 0.0001 | 0.0005 | | 0.0009 | | 0.0009 |  | | |  | |
| 35 | | 1.90 | 0.0002 | 0.0119 | | 0.0182 | | 0.0208 |  | | |  | |
| 206 | | 1.85 | 0.002 | 0.0014 | | 0.0019 | | 0.0022 |  | | |  | |
| 36* | | 1.78 | 0.004 | 0.0010 | | 0.0017 | | 0.0030 |  | | |  | |
| Pentanol | | 1.77 | 0.0004 | 0.0014 | | 0.0021 | | 0.0023 | balsamic, sweet | | |  | |
| 2-Phenyl-2-propanol | | 1.77 | 0.0001 | 0.0005 | | 0.0018 | | 0.0020 | green, sweet, earthy | | |  | |
| 200 | | 1.74 | 0.0009 | 0.0011 | | 0.0020 | | 0.0022 |  | | |  | |
| 31* | | 1.72 | 0.001 | 0.0013 | | 0.0032 | | 0.0039 |  | | |  | |
| 26 | | 1.46 | 0.0001 | 0.0009 | | 0.0018 | | 0.0016 |  | | |  | |
| 2,4-Di-tert-butylphenol | | 1.43 | 0.0008 | 0.0214 | | 0.0327 | | 0.0308 |  | | | antioxidant^5^ | |
| α-Terpineol | | 1.42 | 0.0046 | 0.0018 | | 0.0036 | | 0.0038 | citurs, terpeney, woody | | | hypotensive^33^ gastroprotective^74^ analgesic^32^ antibacterial^31^ antiviral^34^ | |
| 72 | | 1.41 | 0.001 | 0.0012 | | 0.0021 | | 0.0019 |  | | |  | |
| 2,3-Dimethylhexane | | 1.40 | 0.0001 | 0.0013 | | 0.0027 | | 0.0024 |  | | |  | |
| 67 | | 1.35 | 0.004 | 0.0003 | | 0.0005 | | 0.0006 |  | | |  | |
| Sabina ketone | | 1.34 | 0.0001 | 0.0005 | | 0.0010 | | 0.0008 |  | | |  | |
| 204 | | 1.30 | 0.0001 | 0.0071 | | 0.0134 | | 0.0108 |  | | |  | |
| Methyl isobutyl ketone | | 1.28 | 0.0004 | 0.0005 | | 0.0009 | | 0.0008 | herbal, fruity | | |  | |
| o-Xylene | | 1.26 | 0.003 | 0.0016 | | 0.0029 | | 0.0027 | geranium | | |  | |
| (2*E*)-Undecenal | | 1.25 | 0.002 | 0.0012 | | 0.0022 | | 0.0019 | fruity, green | | | antileishmanial^16^ | |
| Geraniol | | 1.24 | 0.006 | 0.0041 | | 0.0073 | | 0.0082 | floral, rose | | | antimicrobial, neuroprotective  anti-inflammatory, antioxidant^75^ | |
| Isoamyl alcohol | | 1.24 | 0.0002 | 0.0007 | | 0.0013 | | 0.0011 | alocholic, banana | | |  | |
| 184 | | 1.24 | 0.01 | 0.0006 | | 0.0009 | | 0.0009 |  | | |  | |
| 2-Ethyl-3,5-dimethylpyrazine | | 1.21 | 0.003 | 0.0003 | | 0.0013 | | 0.0019 | roasted, coffee | | |  | |
| Tetradecanamide | | 1.21 | 0.02 | 0.0066 | | 0.0116 | | 0.0131 |  | | |  | |
| 2,4-Dimethyl-1-heptene | | 1.20 | 0.003 | 0.0020 | | 0.0033 | | 0.0031 |  | | |  | |
| 41 | | 1.16 | 0.0005 | 0.0033 | | 0.0063 | | 0.0051 |  | | |  | |
| Butyl p-toluate | | 1.13 | 0.006 | 0.0014 | | 0.0018 | | 0.0017 |  | | |  | |
| 48 | | 1.12 | 0.002 | 0.0012 | | 0.0019 | | 0.0016 |  | | |  | |
| 56 | | 1.09 | 0.0005 | 0.0007 | | 0.0014 | | 0.0011 |  | | |  | |
| 97 | | 1.04 | 0.0004 | 0.0014 | | 0.0020 | | 0.0017 |  | | |  | |
| **2014** |  |  | | |  | |  | | |  |  | |  |
| 4-Phenyl-3-buten-2-one | | 2.52 | 0.0001 | 0.0003 | | - | | - | fruity, spice | | |  | |
| *m*-tert-Butylphenol | | 2.52 | 0.0001 | 0.0015 | | 0.0004 | | 0.0002 |  | | |  | |
| p-tert-Butylphenol | | 2.41 | 0.0001 | 0.0016 | | 0.0006 | | 0.0004 | earthy, leathery | | |  | |
| 142 | | 2.29 | 0.0001 | 0.0006 | | - | | - |  | | |  | |
| Indane | | 2.29 | 0.0001 | 0.0010 | | 0.0003 | | 0.0004 |  | | |  | |
| Fokienol | | 2.28 | 0.0001 | 0.0056 | | 0.0012 | | 0.0010 |  | | |  | |
| 1-Nitro-2-phenylethane | | 2.08 | 0.0001 | 0.0092 | | 0.0136 | | 0.0123 | floral, spice | | | cardioprotective^45^ | |
| (*E*)-Anethole | | 2.02 | 0.0001 | 0.0009 | | - | | - | sweet, anise | | | antibacterial^31^ anti-inflammatory^76^ antioxidant^77^ | |
| Butyl propanoate | | 2.01 | 0.0001 | 0.0357 | | 0.0014 | | 0.0044 | sweet, earthy, fruity | | |  | |
| (*Z*)-Methyl jasmonate | | 1.88 | 0.0003 | 0.0152 | | 0.0053 | | 0.0037 | floral, jasmine | | | anticancer^8^ anti-inflammatory, antioxidant, neuroprotective, antistress^35^ | |
| 2-Butoxyethanol | | 1.86 | 0.0002 | 0.0216 | | 0.0009 | | 0.0009 |  | | |  | |
| δ-Decalactone | | 1.86 | 0.0001 | 0.0057 | | 0.0015 | | 0.0023 | coconut, peach | | |  | |
| (*Z*)-Methyl *epi*-jasmonate | | 1.82 | 0.0002 | 0.0025 | | 0.0010 | | 0.0009 | sweet, floral | | |  | |
| 168* | | 1.79 | 0.003 | 0.0007 | | 0.0004 | | 0.0002 |  | | |  | |
| 230 | | 1.73 | 0.0002 | 0.0008 | | 0.0003 | | 0.0004 |  | | |  | |
| Jasmine lactone | | 1.66 | 0.0001 | 0.0488 | | 0.0050 | | 0.0041 | jasmine, fruity | | |  | |
| 131 | | 1.59 | 0.003 | 0.0005 | | 0.0002 | | 0.0001 |  | | |  | |
| 211* | | 1.48 | 0.003 | 0.0010 | | 0.0002 | | - |  | | |  | |
| Benzyl acetate | | 1.38 | 0.0001 | 0.0036 | | 0.0005 | | 0.0008 | sweet, floral, fruity | | | antifungal^18^ | |
| Viridene | | 1.36 | 0.0001 | 0.0031 | | 0.0005 | | 0.0010 |  | | |  | |
| Indene | | 1.33 | 0.002 | 0.0005 | | 0.0003 | | 0.0004 |  | | |  | |
| Benzyl alcohol | | 1.21 | 0.002 | 0.0026 | | 0.0009 | | 0.0013 | floral, cherry | | | antioxidant^20^ | |
| Indole | | 1.15 | 0.004 | 0.1062 | | 0.0258 | | 0.0532 | fecal, mothball, floral | | | antibacterial^7^ antifungal^18^ | |
| 191 | | 1.14 | 0.0001 | 0.0015 | | - | | - |  | | |  | |
| γ-Octanolactone | | 1.13 | 0.02 | 0.0007 | | 0.0002 | | 0.0003 | sweet, coconut, waxy | | |  | |
| **2014/2016** |  |  | | |  | |  | | |  |  | |  |
| 130 | | 1.24 | 0.0002 | 0.0019 | | 0.0010 | | 0.0036 |  | | |  | |
| Menthol | | 1.18 | 0.0001 | 0.0035 | | 0.0007 | | 0.0019 | peppermint, cooling | | | antibacterial^31^ decongestant^46^ cardioprotective^33^ analgesic^32^ | |
| 3,4-Diethyl-1,1'-biphenyl | | 1.14 | 0.0002 | 0.0017 | | 0.0004 | | 0.0010 |  | | |  | |
| **2014/2015** |  |  | | |  | |  | | |  |  | |  |
| 2,3,5-Trimethylhexane | | 2.12 | 0.0001 | 0.0015 | | 0.0010 | | 0.0004 |  | | |  | |
| 180 | | 1.22 | 0.006 | 0.0002 | | 0.0002 | | - |  | | |  | |
| 80 | | 1.17 | 0.01 | 0.0011 | | 0.0014 | | 0.0004 |  | | |  | |
| **2015** |  |  | | |  | |  | | |  |  | |  |
| Methyl 4-methyl benzoate | | 1.04 | 0.0001 | 0.0004 | | 0.0010 | | 0.0007 | sweet, anise, floral | | |  | |
| 28 | | 1.02 | 0.0001 | 0.0016 | | 0.0029 | | 0.0022 |  | | |  | |

^a^Aroma information obtained from the Good Scents Company^39^ unless otherwise noted. *Compound is affected by more than one environmental factor. Numbers in the compound column refer to unknown compounds.

**Figure S1.** PC1 vs PC4 score plot of Fujian tea.

1. Cheriyan, B., Kadhirvelu, P., Nadipelly, J., Shanmugasundaram, J., Sayeli, V., and Subramanian, V. (2017). Anti-nociceptive effect of 7-methoxy coumarin from Eupatorium Triplinerve vahl (Asteraceae). *Pharmacognosy Magazine* 13**,** 81-84.

2. Mousavi, S.H., Davari, A.-S., Iranshahi, M., Sabouri-Rad, S., and Tayarani Najaran, Z. (2015). Comparative analysis of the cytotoxic effect of 7-prenyloxycoumarin compounds and herniarin on MCF-7 cell line. *Avicenna Journal of Phytomedicine* 5**,** 520-530.

3. Silván, A.M., Abad, M.J., Bermejo, P., Sollhuber, M., and Villar, A. (1996). Antiinflammatory Activity of Coumarins from Santolina oblongifolia. *Journal of Natural Products* 59**,** 1183-1185.

4. Joshi, R., and Gulati, A. (2015). Fractionation and identification of minor and aroma-active constituents in Kangra orthodox black tea. *Food Chemistry* 167**,** 290-298.

5. Choi, S.J., Kim, J.K., Kim, H.K., Harris, K., Kim, C.-J., Park, G.G., Park, C.-S., and Shin, D.-H. (2013). 2,4-Di-tert-butylphenol from Sweet Potato Protects Against Oxidative Stress in PC12 Cells and in Mice. *Journal of Medicinal Food* 16**,** 977-983.

6. Yang, J.-Y., Kim, M.-G., Park, J.-H., Hong, S.-T., and Lee, H.-S. (2014). Evaluation of benzaldehyde derivatives from *Morinda officinalis* as anti-mite agents with dual function as acaricide and mite indicator. *Scientific Reports* 4**,** 7149.

7. Muroi, H., and Kubo, I. (1993). Combination Effects of Antibacterial Compounds in Green Tea Flavor Against *Streptococcus-mutans*. *Journal of Agricultural and Food Chemistry* 41**,** 1102-1105.

8. Tong, Q.S., Jiang, G.S., Zheng, L.D., Tang, S.T., Cai, J.B., Liu, Y., Zeng, F.Q., and Dong, J.H. (2008). Natural jasmonates of different structures suppress the growth of human neuroblastoma cell line SH-SY5Y and its mechanisms. *Acta Pharmacologica Sinica* 29**,** 861-869.

9. Fidyt, K., Fiedorowicz, A., Strzadala, L., and Szumny, A. (2016). beta-caryophyllene and beta-caryophyllene oxide-natural compounds of anticancer and analgesic properties. *Cancer Medicine* 5**,** 3007-3017.

10. Bahi, A., Al Mansouri, S., Al Memari, E., Al Ameri, M., Nurulain, S.M., and Ojha, S. (2014). beta-Caryophyllene, a CB2 receptor agonist produces multiple behavioral changes relevant to anxiety and depression in mice. *Physiology & Behavior* 135**,** 119-124.

11. Klauke, A.L., Racz, I., Pradier, B., Markert, A., Zimmer, A.M., Gertsch, J., and Zimmer, A. (2014). The cannabinoid CB2 receptor-selective phytocannabinoid beta-caryophyllene exerts analgesic effects in mouse models of inflammatory and neuropathic pain. *European Neuropsychopharmacology* 24**,** 608-620.

12. Guerrini, A., Sacchetti, G., Grandini, A., Spagnoletti, A., Asanza, M., and Scalvenzi, L. (2016). Cytotoxic Effect and TLC Bioautography-Guided Approach to Detect Health Properties of Amazonian Hedyosmum sprucei Essential Oil. *Evidence-Based Complementary and Alternative Medicine***,** 8.

13. Rossi, D., Guerrini, A., Maietti, S., Bruni, R., Paganetto, G., Poli, F., Scalvenzi, L., Radice, M., Saro, K., and Sacchetti, G. (2011). Chemical fingerprinting and bioactivity of Amazonian Ecuador *Croton lechleri* Mull. Arg. (Euphorbiaceae) stem bark essential oil: A new functional food ingredient? *Food Chemistry* 126**,** 837-848.

14. Resende, D.B., Martins, H.H.D.A., Souza, T.B.D., Carvalho, D.T., Piccoli, R.H., Schwan, R.F., and Dias, D.R. (2017). Synthesis and in vitro evaluation of peracetyl and deacetyl glycosides of eugenol, isoeugenol and dihydroeugenol acting against food-contaminating bacteria. *Food Chemistry* 237**,** 1025-1029.

15. Marteau, C., Guitard, R., Penverne, C., Favier, D., Nardello-Rataj, V., and Aubry, J.-M. (2016). Boosting effect of ortho-propenyl substituent on the antioxidant activity of natural phenols. *Food Chemistry* 196**,** 418-427.

16. Donega, M.A., Mello, S.C., Moraes, R.M., Jain, S.K., Tekwani, B.L., and Cantrell, C.L. (2014). Pharmacological Activities of Cilantroʼs Aliphatic Aldehydes against Leishmania donovani. *Planta Med* 80**,** 1706-1711.

17. Astudillo, A.M., Meana, C., Guijas, C., Pereira, L., Lebrero, P., Balboa, M.A., and Balsinde, J. (2018). Occurance and biological activity of palmitoleic acid isomers in phagocytic cells. *Journal of Lipid Research* 59**,** 237-249.

18. Nidiry, E.S., and Babu, C.S.B. (2005). Antifungal activity of tuberose absolute and some of its constituents. *Phytotherapy Research* 19**,** 447-449.

19. Kumar, D., and Kumar, S. (2017). Isolation and Characterization of Bioactive Phenolic Compounds from Abies Pindrow Aerial Parts. *Pharmaceutical Chemistry Journal* 51**,** 205-210.

20. Shiratsuchi, H., Chang, S., Wei, A., El-Ghorab, A.H., and Shibamoto, T. (2012). Biological activities of low-molecular weight compounds found in foods and plants. *Journal of Food and Drug Analysis* 20**,** 359-365.

21. Sun, S., Du, G.J., Qi, L.W., Williams, S., Wang, C.Z., and Yuan, C.S. (2010). Hydrophobic constituents and their potential anticancer activities from Devil's Club (Oplopanax horridus Miq.). *Journal of Ethnopharmacology* 132**,** 280-285.

22. Tung, Y.T., Yen, P.L., Lin, C.Y., and Chang, S.T. (2010). Anti-inflammatory activities of essential oils and their constituents from different provenances of indigenous cinnamon (Cinnamomum osmophloeum) leaves. *Pharmaceutical Biology* 48**,** 1130-1136.

23. Russo, E.B. (2011). Taming THC: potential cannabis synergy and phytocannabinoid-terpenoid entourage effects. *British Journal of Pharmacology* 163**,** 1344-1364.

24. Koziol, A., Stryjewska, A., Librowski, T., Salat, K., Gawal, M., Moniczewski, A., and Lochynski, S. (2014). An Overview of the Pharmacological Properties and Potential Applications of Natural Monoterpenes. *Mini Reviews in Medicinal Chemistry* 14**,** 1156-1168.

25. Morales-López, J., Centeno-Álvarez, M., Nieto-Camacho, A., López, M.G., Pérez-Hernández, E., Pérez-Hernández, N., and Fernández-Martínez, E. (2017). Evaluation of antioxidant and hepatoprotective effects of white cabbage essential oil. *Pharmaceutical Biology* 55**,** 233-241.

26. Bellincontro, A., Matarese, F., D'onofrio, C., Accordini, D., Tosi, E., and Mencarelli, F. (2016). Management of postharvest grape withering to optimise the aroma of the final wine: A case study on Amarone. *Food Chemistry* 213**,** 378-387.

27. Jeong, J.B., Hong, S.C., Jeong, H.J., and Koo, J.S. (2011). Anti-inflammatory effect of 2-methoxy-4-vinylphenol via the suppression of NF-κB and MAPK activation, and acetylation of histone H3. *Archives of Pharmacal Research* 34**,** 2109-2116.

28. Afoulous, S., Ferhout, H., Raoelison, E.G., Valentin, A., Moukarzel, B., Couderc, F., and Bouajila, J. (2011). Helichrysum gymnocephalum Essential Oil: Chemical Composition and Cytotoxic, Antimalarial and Antioxidant Activities, Attribution of the Activity Origin by Correlations. *Molecules* 16**,** 8273-8291.

29. Takei, M., Umeyama, A., and Arihara, S. (2006). T-cadinol and calamenene induce dendritic cells from human monocytes and drive Th1 polarization. *European Journal of Pharmacology* 537**,** 190-199.

30. Kim, G., Kim, J.-E., Kang, M.-J., Jang, A.-R., Kim, Y.R., Kim, S., Chang, K.-T., Hong, J.J., and Park, J.-H. (2017). Inhibitory effect of 1-tetradecanol on Helicobacter pylori-induced production of interleukin-8 and vascular endothelial growth factor in gastric epithelial cells. *Molecular Medicine Reports* 16**,** 9573-9578.

31. Iscan, G. (2017). Antibacterial and Anticandidal Activities of Common Essential Oil Constituents. *Records of Natural Products* 11**,** 374-388.

32. Guimarães, A.G., Quintans, J.S.S., and Quintans-Júnior, L.J. (2013). Monoterpenes with Analgesic Activity—A Systematic Review. *Phytotherapy Research* 27**,** 1-15.

33. Santos, M.R.V., Moreira, F.V., Fraga, B.P., Souza, D.P.D., Bonjardim, L.R., and Quintans-Junior, L.J. (2011). Cardiovascular effects of monoterpenes: a review. *Revista Brasileira de Farmacognosia* 21**,** 764-771.

34. Astani, A., Reichling, J., and Schnitzler, P. (2010). Comparative study on the antiviral activity of selected monoterpenes derived from essential oils. *Phytotherapy Research* 24**,** 673-679.

35. Adebesin, A., Ajayi Abayomi, M., Olonode Elizabeth, O., Omorogbe, O., and Umukoro, S. (2017). Methyl Jasmonate Ameliorates Unpredictable Chronic Mild Stress‐Induced Behavioral and Biochemical Alterations in Mouse Brain. *Drug Development Research* 78**,** 381-389.

36. Battinelli, L., Daniele, C., Cristiani, M., Bisignano, G., Saija, A., and Mazzanti, G. (2006). In vitro antifungal and anti-elastase activity of some aliphatic aldehydes from *Olea europaea* L. fruit. *Phytomedicine* 13**,** 558-563.

37. Fujita, K.-I., Chavasiri, W., and Kubo, I. (2015). Anti-Salmonella Activity of Volatile Compounds of Vietnam Coriander. *Phytotherapy Research* 29**,** 1081-1087.

38. Buhrer, C., Bahr, S., Siebert, J., Wettstein, R., Geffers, C., and Obladen, M. (2002). Use of 2% 2-phenoxyethanol and 0.1% octenidine as antiseptic in premature newborn infants of 23-26 weeks gestation. *Journal of Hospital Infection* 51**,** 305-307.

39. Perflavory (2015). "The Good Scents Company". (Oak Creek, WI).

40. Shan, M.Q., Qian, Y., Yu, S., Guo, S.C., Zhang, L., Ding, A.W., and Wu, Q.N. (2016). Anti-inflammatory effect of volatile oil from *Schizonepeta tenuifolia* on carrageenin-induced pleurisy in rats and its application to study of appropriate harvesting time coupled with multi-attribute comprehensive index method. *Journal of Ethnopharmacology* 194**,** 580-586.

41. Odeh, I., Abu-Lafi, S., and Al-Najjar, I. (2014). Determination of Unifloral Honey Volatiles from Centaurea iberica and Zizyphus spinachristi by Solid-Phase Microextraction and Gas Chromatography-Mass Spectrometry. *Acta Chromatographica* 26**,** 485-493.

42. Boskabady, M.H., and Farkhondeh, T. (2016). Antiinflammatory, Antioxidant, and Immunomodulatory Effects of Crocus sativus L. and its Main Constituents. *Phytotherapy Research* 30**,** 1072-1094.

43. Carradori, S., Chimenti, P., Fazzari, M., Granese, A., and Angiolella, L. (2016). Antimicrobial activity, synergism and inhibition of germ tube formation by Crocus sativus-derived compounds against Candida spp. *Journal of Enzyme Inhibition and Medicinal Chemistry* 31**,** 189-193.

44. Nieto-Bobadilla, M.S., Siepmann, F., Djouina, M., Dubuquoy, L., Tesse, N., Willart, J.F., Dubreuil, L., Siepmann, J., and Neut, C. (2015). Controlled delivery of a new broad spectrum antibacterial agent against colitis: In vitro and in vivo performance. *European Journal of Pharmaceutics and Biopharmaceutics* 96**,** 152-161.

45. Vasconcelos, T.B.D., Ribeiro-Filho, H.V., Lahlou, S., Pereira, J.G.D.C., Oliveira, P.S.L.D., and Magalhães, P.J.C. (2018). Vasodilator effects and putative guanylyl cyclase stimulation by 2-nitro-1-phenylethanone and 2-nitro-2-phenyl-propane-1,3-diol on rat aorta. *European Journal of Pharmacology* 830**,** 105-114.

46. Eccles, R. (2003). Menthol: Effects on nasal sensation of airflow and the drive to breathe. *Current Allergy and Asthma Reports* 3**,** 210-214.

47. Brul, S., and Coote, P. (1999). Preservative agents in foods: Mode of action and microbial resistance mechanisms. *International Journal of Food Microbiology* 50**,** 1-17.

48. Kako, H., Kobayashi, Y., and Yokogoshi, H. (2012). Dopamine release from rat pheochromocytoma (PC12) cells and rat brain striata induced by a series of straigh carbon chain aldehydes with variations in carbon chain length and functional groups. *European Journal of Pharmacology* 691**,** 86-92.

49. Tse, G., Yeo, J.M., Tse, V., Kwan, J., and Sun, B. (2016). Gap junction inhibition by heptanol increases ventricular arrhythmogenicity by reducing conduction velocity without affecting repolarization properties or myocardial refractoriness in Langendorff-perfused mouse hearts. *Molecular Medicine Reports* 14**,** 4069-4074.

50. Furtado Kelly, S., De Oliveira Andrade, F., Campos, A., Rosim Mariana, P., Vargas‐Mendez, E., Henriques, A., De Conti, A., Scolastici, C., Barbisan Luis, F., Carvalho Robson, F., and Moreno Fernando, S. (2016). β‐ionone modulates the expression of miRNAs and genes involved in the metastatic phenotype of microdissected persistent preneoplastic lesions in rats submitted to hepatocarcinogenesis. *Molecular Carcinogenesis* 56**,** 184-196.

51. Oz, M., Lozon, Y., Sultan, A., Yang, K.-H.S., and Galadari, S. (2015). Effects of monoterpenes on ion channels of excitable cells. *Pharmacology & Therapeutics* 152**,** 83-97.

52. Salakhutdinov Nariman, F., Volcho Konstantin, P., and Yarovaya Olga, I. (2017). Monoterpenes as a renewable source of biologically active compounds. *Pure and Applied Chemistry* 89**,** 1105.

53. Moro, I.J., Gondo, G.D.G.A., Pierri, E.G., Pietro, R.C.L.R., Soares, C.P., De Sousa, D.P., and Dos Santos, A.G. (2017). Evaluation of antimicrobial, cytotoxic and chemopreventive activies of carvone and its derivatives. *Brazilian Journal of Pharmaceutical Sciences* 53**,** e00076.

54. Ianaro, A., Ialenti, A., Maffia, P., Di Meglio, P., Di Rosa, M., and Santoro, M.G. (2003). Anti-Inflammatory Activity of 15-Deoxy-Δ^12,14^-PGJ_2_ and 2-Cyclopenten-1-one: Role of the Heat Shock Response. *Molecular Pharmacology* 64**,** 85-93.

55. Miron, D., Battisti, F., Silva, F.K., Lana, A.D., Pippi, B., Casanova, B., Gnoatto, S., Fuentefria, A., Mayorga, P., and Schapoval, E.E.S. (2014). Antifungal activity and mechanism of action of monoterpenes against dermatophytes and yeasts. *Revista Brasileira de Farmacognosia* 24**,** 660-667.

56. Higashi, Y., Kiuchi, T., and Furuta, K. (2010). Efficacy and safety profile of topical methyl salicylate and menthol patch in adult patients with mild to moderate muscle strain: A randomized, double-blind, parallel-group, placebo-controlled, multicenter study. *Clinical Therapeutics* 32**,** 34-43.

57. Chowdhury, L., Croft, C.J., Goel, S., Zaman, N., Tai, A.C.-S., Walch, E.M., Smith, K., Page, A., Shea, K.M., Hall, C.D., Jishkariani, D., Pillai, G.G., and Hall, A.C. (2016). Differential Potency of 2,6-Dimethylcyclohexanol Isomers for Positive Modulation of GABA_A_ Receptor Currents. *Journal of Pharmacology and Experimental Therapeutics* 357**,** 570-579.

58. Kima, J., Seo, J., Bae, M., Yoo, J., Bang, M., Cho, S., and Park, D. (2016). Antimicrobial constituents from Allium hookeri Root. *Natural Product Communications* 11**,** 237-238.

59. Park, K.H., and Lee, M.W. (2012). Anti-oxidative, anti-inflammatory and whitening effects of phenolic compounds from Bambusae Caulis in Liquamen. *Natural Product Research* 26**,** 1687-1691.

60. Alves Naiane Ferraz, B., Queiroz Thyago, M., Almeida Travassos, R., Magnani, M., and Andrade Braga, V. (2016). Acute Treatment with Lauric Acid Reduces Blood Pressure and Oxidative Stress in Spontaneously Hypertensive Rats. *Basic & Clinical Pharmacology & Toxicology* 120**,** 348-353.

61. Wang, J., Lu, J., Xie, X., Xiong, J., Huang, N., Wei, H., Jiang, S., and Peng, J. (2018). Blend of organic acids and medium chain fatty acids prevents the inflammatory response and intestinal barrier dysfunction in mice challenged with enterohemorrhagic Escherichia coli O157:H7. *International Immunopharmacology* 58**,** 64-71.

62. Vahid, H., Rakhshandeh, H., and Ghorbani, A. (2017). Antidiabetic properties of Capparis spinosa L. and its components. *Biomedicine & Pharmacotherapy* 92**,** 293-302.

63. Egan, D., O'kennedy, R., Moran, E., Cox, D., Prosser, E., and Thornes, R.D. (1990). The Pharmacology, Metabolism, Analysis, and Applications of Coumarin and Coumarin-Related Compounds. *Drug Metabolism Reviews* 22**,** 503-529.

64. Song, Y., Sun, R., Ji, Z., Li, X., Fu, Q., and Ma, S. (2018). Perilla aldehyde attenuates CUMS-induced depressive-like behaviors via regulating TXNIP/TRX/NLRP3 pathway in rats. *Life Sciences* 206**,** 117-124.

65. Do Nascimento, K.F., Moreira, F.M.F., Alencar Santos, J., Kassuya, C.a.L., Croda, J.H.R., Cardoso, C.a.L., Vieira, M.D.C., Góis Ruiz, A.L.T., Ann Foglio, M., De Carvalho, J.E., and Formagio, A.S.N. (2018). Antioxidant, anti-inflammatory, antiproliferative and antimycobacterial activities of the essential oil of Psidium guineense Sw. and spathulenol. *Journal of Ethnopharmacology* 210**,** 351-358.

66. Nguyen, L.T., Myslivečková, Z., Szotáková, B., Špičáková, A., Lněničková, K., Ambrož, M., Kubíček, V., Krasulová, K., Anzenbacher, P., and Skálová, L. (2017). The inhibitory effects of β-caryophyllene, β-caryophyllene oxide and α-humulene on the activities of the main drug-metabolizing enzymes in rat and human liver in vitro. *Chemico-Biological Interactions* 278**,** 123-128.

67. Anjali, Pathak, D., and Singh, D. (2016). Quinoline: A Diverse Therapeutic Agent. *International Journal of Pharmaceutical Sciences and Research* 7**,** 1-13.

68. Santos, N.C., Figueira-Coelho, J., Martins-Silva, J., and Saldanha, C. (2003). Multidisciplinary utilization of dimethyl sulfoxide: pharmacological, cellular, and molecular aspects. *Biochemical Pharmacology* 65**,** 1035-1041.

69. Horishita, T., and Harris, R.A. (2008). *n*-Alcohols Inhibit Voltage-Gated Na^+^ Channels Expressed in *Xenopus* Oocytes. *Journal of Pharmacology and Experimental Therapeutics* 326**,** 270-277.

70. Abuhamdah, S., Talji, D., Abuirmeileh, N., Bahnassi, A., Salahat, I., and Abuirmeileh, A. (2017). Behavioral and Neurochemical Alterations Induced by Vanillin in a Mouse Model of Alzheimer's Disease. *International Journal of Pharmacology* 13**,** 573-582.

71. González-Ramírez, A.E., González-Trujano, M.E., Orozco-Suárez, S.A., Alvarado-Vásquez, N., and López-Muñoz, F.J. (2016). Nerol alleviates pathologic markers in the oxazolone-induced colitis model. *European Journal of Pharmacology* 776**,** 81-89.

72. Popova, A.A., Koksharova, O.A., Lipasova, V.A., Zaitseva, J.V., Katkova-Zhukotskaya, O.A., Eremina, S.I., Mironov, A.S., Chernin, L.S., and Khmel, I.A. (2014). Inhibitory and Toxic Effects of Volatiles Emitted by Strains of Pseudomonas and Serratia on Growth and Survival of Selected Microorganisms, Caenorhabditis elegans, and Drosophila melanogaster. *BioMed Research International* 2014**,** 11.

73. Nakayama, K., Murata, S., Ito, H., Iwasaki, K., Villareal, M.O., Zheng, Y.-W., Matsui, H., Isoda, H., and Ohkohchi, N. (2017). Terpinen-4-ol inhibits colorectal cancer growth via reactive oxygen species. *Oncology Letters* 14**,** 2015-2024.

74. Souza, R.H.L., Cardoso, M.S.P., Menezes, C.T., Silva, J.P., De Sousa, D.P., and Batista, J.S. (2011). Gastroprotective activity of α-terpineol in two experimental models of gastric ulcer in rats. *Journal of Pharmaceutical Sciences* 19**,** 277-281.

75. Pavan, B., Dalpiaz, A., Marani, L., Beggiato, S., Ferraro, L., Canistro, D., Paolini, M., Vivarelli, F., Valerii, M.C., Comparone, A., De Fazio, L., and Spisni, E. (2018). Geraniol Pharmacokinetics, Bioavailability and Its Multiple Effects on the Liver Antioxidant and Xenobiotic-Metabolizing Enzymes. *Frontiers in Pharmacology* 9.

76. Kim, K.Y., Lee, H.S., and Seol, G.H. (2017). Anti-inflammatory effects of trans-anethole in a mouse model of chronic obstructive pulmonary disease. *Biomedicine & Pharmacotherapy* 91**,** 925-930.

77. Donati, M., Mondin, A., Chen, Z., Miranda, F.M., Do Nascimento, B.B., Schirato, G., Pastore, P., and Froldi, G. (2015). Radical scavenging and antimicrobial activities of Croton zehntneri, Pterodon emarginatus and Schinopsis brasiliensis essential oils and their major constituents: estragole, trans-anethole, β-caryophyllene and myrcene. *Natural Product Research* 29**,** 939-946.
